# Supplementary figures and images for: Four ZIPs contribute to Zn, Fe, Cu and Mn acquisition at the outer root domain
Source: PLoS Genet. 2025 Jul 15;21(7):e1011796. doi: 10.1371/journal.pgen.1011796 (PMC12279101; doi:10.1371/journal.pgen.1011796)

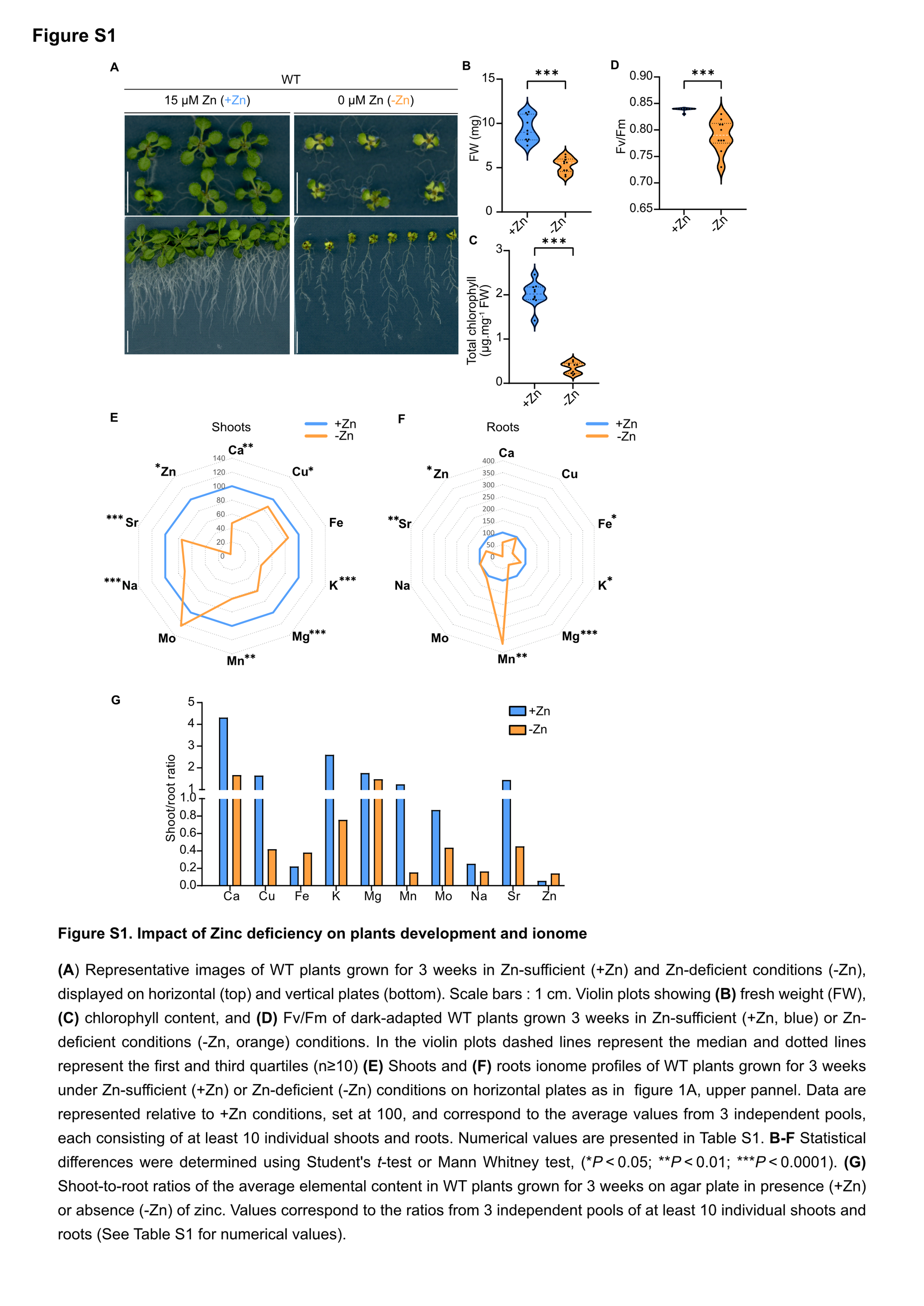

Supplement: S1 Fig — (A) Representative images of WT plants grown for 3 weeks in Zn-sufficient (+Zn) and Zn-deficient conditions (-Zn), displayed on horizontal (top) and vertical plates (bottom). Scale bars: 1 cm. Violin plots showing (B) fresh weight (FW), (C) chlorophyll content, and (D) Fv/Fm of dark-adapted WT plants grown 3 weeks in Zn-sufficient (+Zn, blue) or Zn-deficient conditions (-Zn, orange) conditions. In the violin plots dashed lines represent the median and dotted lines represent the first and third quartiles (n ≥ 10) (E) Shoots and (F) roots ionome profiles of WT plants grown for 3 weeks under Zn-sufficient (+Zn) or Zn-deficient (-Zn) conditions on horizontal plates as in upper panel A. Data are represented relative to +Zn conditions, set at 100, and correspond to the average values from 3 independent pools, each consisting of at least 10 individual shoots and roots. Numerical values are presented in S1 Table. B-F Statistical differences were determined using Student’s t-test or Mann Whitney test, (*P < 0.05; **P < 0.01; ***P < 0.0001). (G) Shoot-to-root ratios of the average elemental content in WT plants grown for 3 weeks on agar plate in presence (+Zn) or absence (-Zn) of zinc. Values correspond to the ratios from 3 independent pools of at least 10 individual shoots and roots (See S1 Table for numerical values). (TIF) [file pgen.1011796.s001.tif]

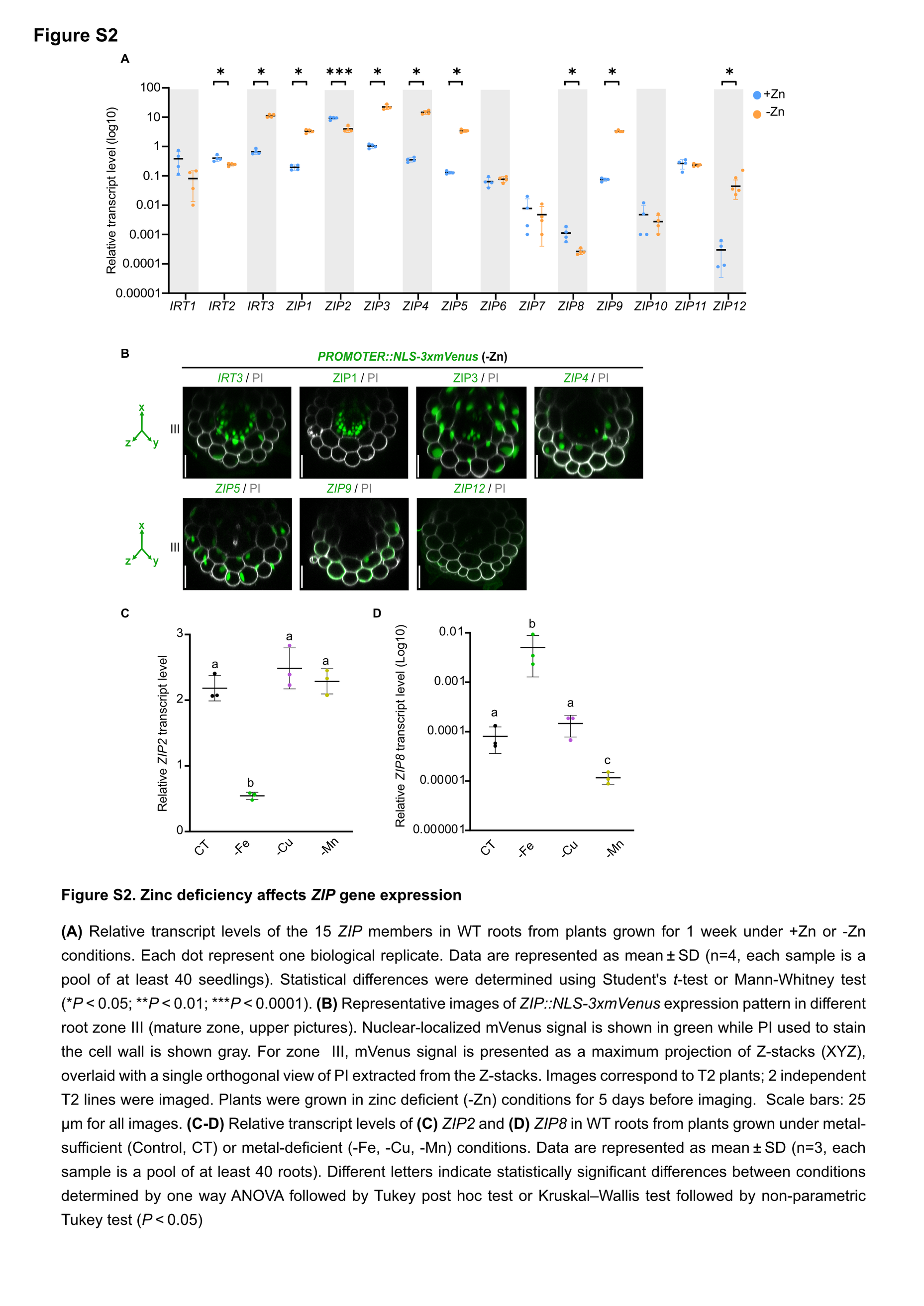

Supplement: S2 Fig — (A) Relative transcript levels of the 15 ZIP members in WT roots from plants grown for 1 week under +Zn or -Zn conditions. Each dot represent one biological replicate. Data are represented as mean ± SD (n = 4, each sample is a pool of at least 40 seedlings). Statistical differences were determined using Student’s t-test or Mann-Whitney test (*P < 0.05; **P < 0.01; ***P < 0.0001). (B) Representative images of ZIP::NLS-3xmVenus expression pattern in differentiated root (zone III). Nuclear-localized mVenus signal is shown in green while PI used to stain the cell wall is shown gray. mVenus signal is presented as a maximum projection of Z-stacks (XYZ), overlaid with a single orthogonal view of PI extracted from the Z-stacks. Images correspond to T2 plants; 2 independent T2 lines were imaged. Plants were grown in zinc deficient (-Zn) conditions for 5 days before imaging. Scale bars: 25 µm for all images. (C-D) Relative transcript levels of (C) ZIP2 and (D) ZIP8 in WT roots from plants grown under metal-sufficient (Control, CT) or metal-deficient (-Fe, -Cu, -Mn) conditions. Data are represented as mean ± SD (n = 3, each sample is a pool of at least 40 roots). Different letters indicate statistically significant differences between conditions determined by one way ANOVA followed by Tukey post hoc test or Kruskal–Wallis test followed by non-parametric Tukey test (P < 0.05). (TIF) [file pgen.1011796.s002.tif]

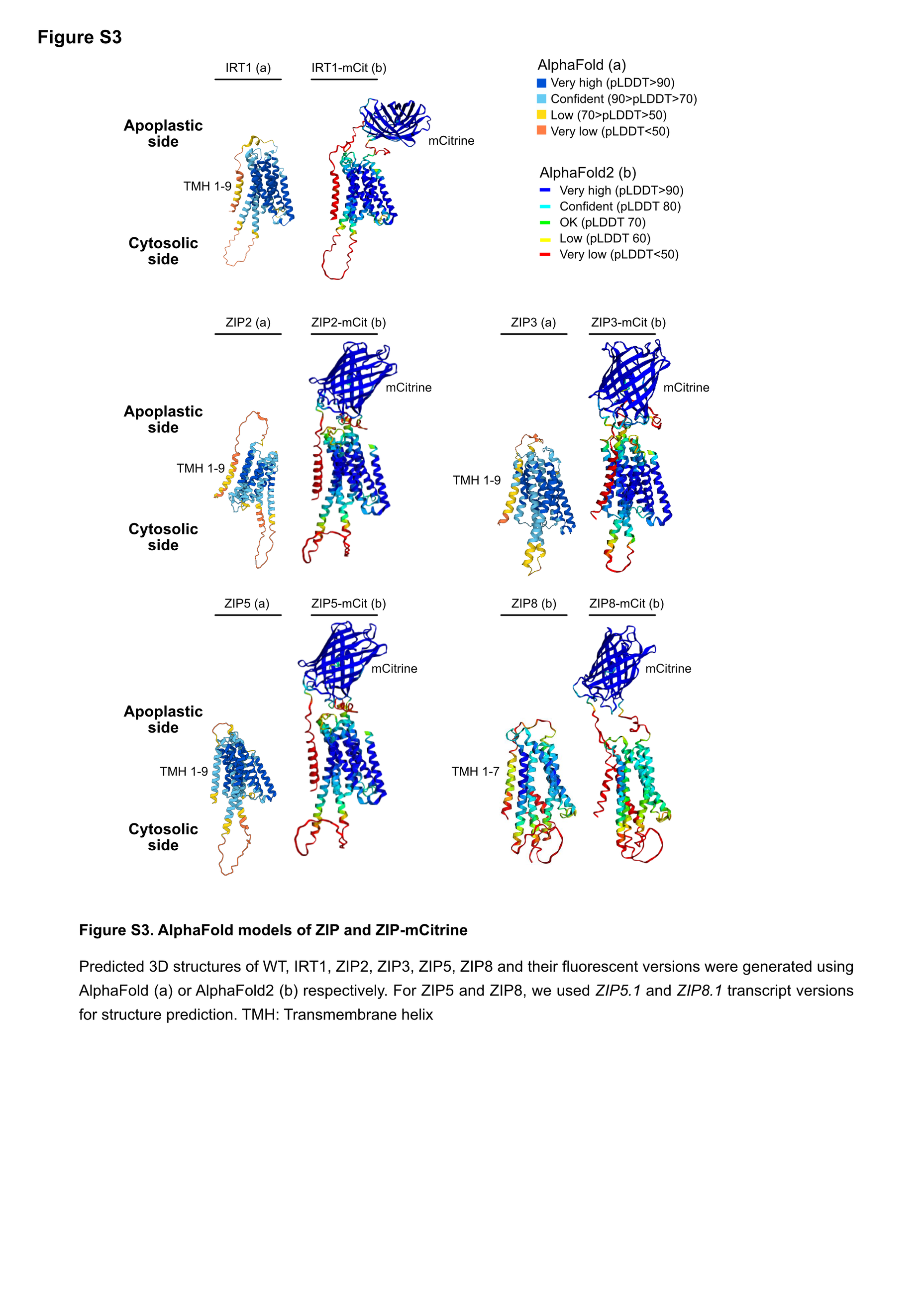

Supplement: S3 Fig — Predicted 3D structures of WT, IRT1, ZIP2, ZIP3, ZIP5, ZIP8 and their fluorescent versions were generated using AlphaFold (a) or AlphaFold2 (b) respectively. For ZIP5 and ZIP8, we used ZIP5.1 and ZIP8.1 transcript versions for structure prediction. TMH: Transmembrane helix. (TIF) [file pgen.1011796.s003.tif]

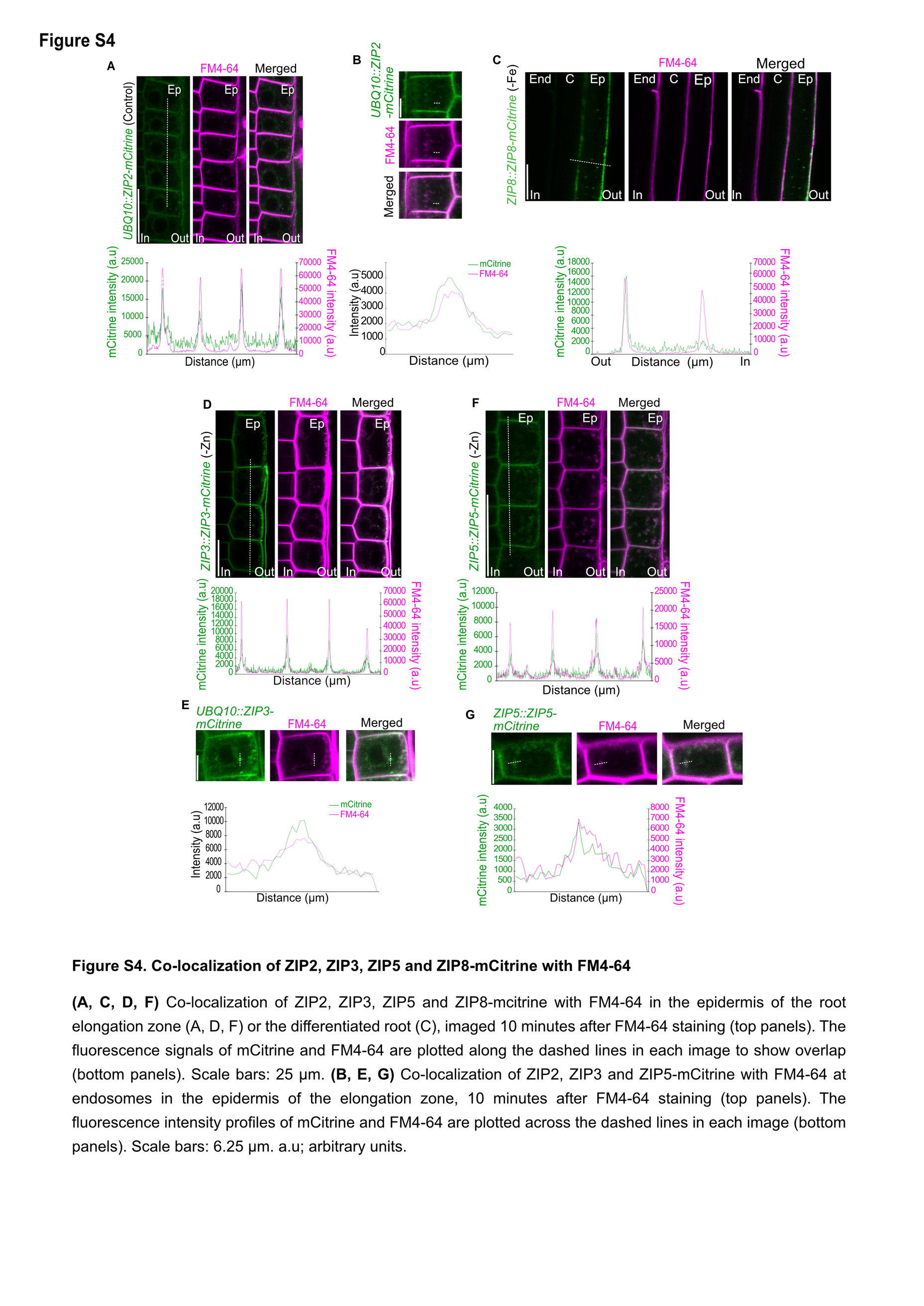

Supplement: S4 Fig — (A, C, D, F) Co-localization of ZIP2, ZIP3, ZIP5 and ZIP8-mcitrine with FM4–64 in the epidermis of the root elongation zone (A, D, F) or the differentiated root (C), imaged 10 minutes after FM4–64 staining (top panels). The fluorescence signals of mCitrine and FM4–64 are plotted along the dashed lines in each image to show overlap (bottom panels). Scale bars: 25 µm. (B, E, G) Co-localization of ZIP2, ZIP3 and ZIP5-mCitrine with FM4–64 at endosomes in the epidermis of the elongation zone, 10 minutes after FM4–64 staining (top panels). The fluorescence intensity profiles of mCitrine and FM4–64 are plotted across the dashed lines in each image (bottom panels). Scale bars: 6.25 µm. a.u; arbitrary units. (TIF) [file pgen.1011796.s004.tif]

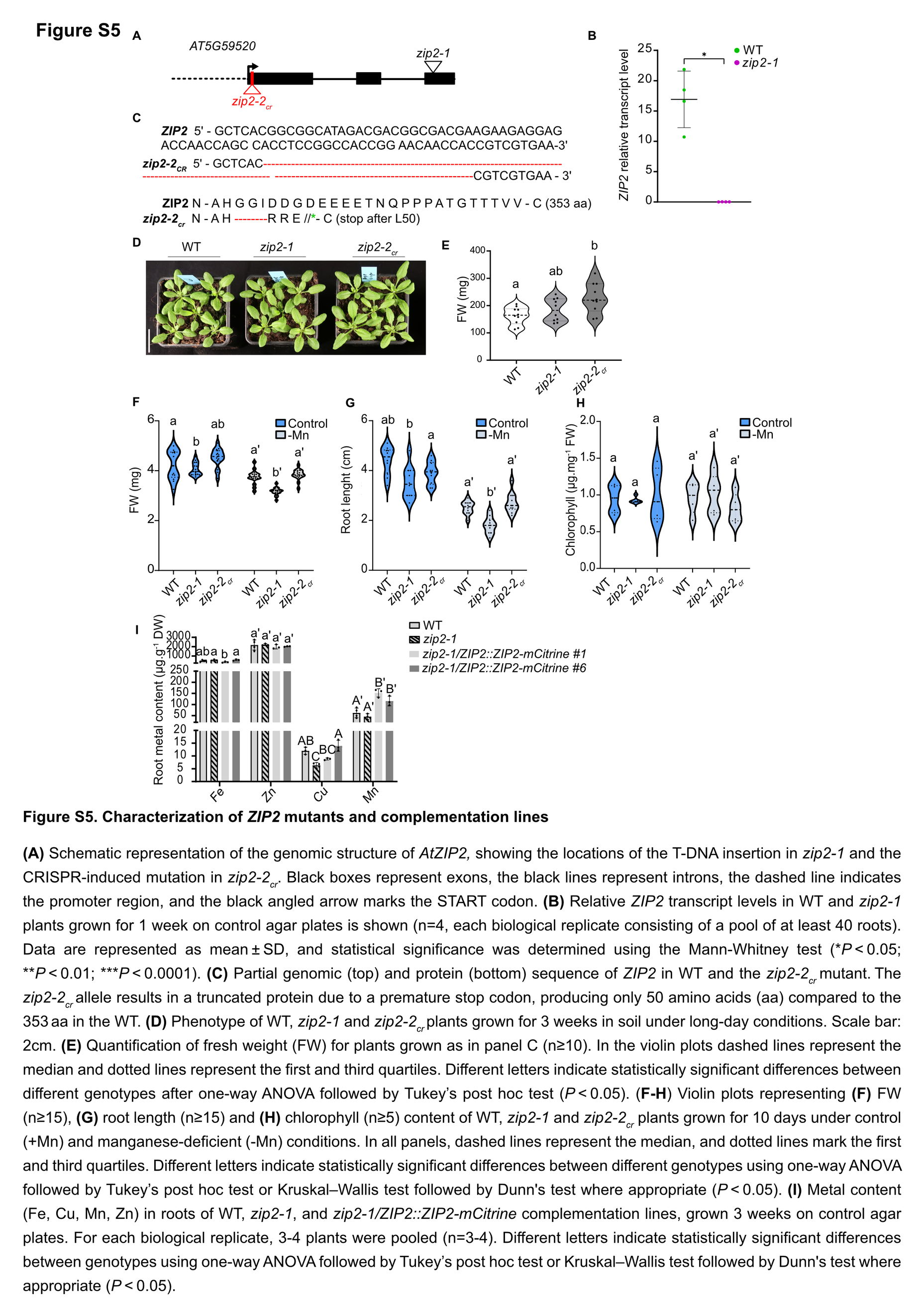

Supplement: S5 Fig — (A) Schematic representation of the genomic structure of AtZIP2, showing the locations of the T-DNA insertion in zip2–1 and the CRISPR-induced mutation in zip2–2cr. Black boxes represent exons, the black lines represent introns, the dashed line indicates the promoter region, and the black angled arrow marks the START codon. (B) Relative ZIP2 transcript levels in WT and zip2–1 plants grown for 1 week on control agar plates is shown (n = 4, each biological replicate consisting of a pool of at least 40 roots). Data are represented as mean ± SD, and statistical significance was determined using the Mann-Whitney test (*P < 0.05; **P < 0.01; ***P < 0.0001). (C) Partial genomic (top) and protein (bottom) sequence of ZIP2 in WT and the zip2–2cr mutant. The zip2–2cr allele results in a truncated protein due to a premature stop codon, producing only 50 amino acids (aa) compared to the 353 aa in the WT. (D) Phenotype of WT, zip2–1 and zip2–2cr plants grown for 3 weeks in soil under long-day conditions. Scale bar: 2 cm. (E) Quantification of fresh weight (FW) for plants grown as in panel C (n ≥ 10). In the violin plots dashed lines represent the median, and dotted lines represent the first and third quartiles. Different letters indicate statistically significant differences between different genotypes after one-way ANOVA followed by Tukey’s post hoc test (P < 0.05). (F-H) Violin plots representing (F) FW (n ≥ 15), (G) root length (n ≥ 15) and (H) chlorophyll (n ≥ 5) content of WT, zip2–1 and zip2–2cr plants grown for 10 days under control (+Mn) and manganese-deficient (-Mn) conditions. In all panels, dashed lines represent the median, and dotted lines mark the first and third quartiles. Different letters indicate statistically significant differences between different genotypes using one-way ANOVA followed by Tukey’s post hoc test or Kruskal–Wallis test followed by Dunn’s test where appropriate (P < 0.05). (I) Metal content (Fe, Cu, Mn, Zn) in roots of WT, zip2–1, and zip2–1/ZI [file pgen.1011796.s005.tif]

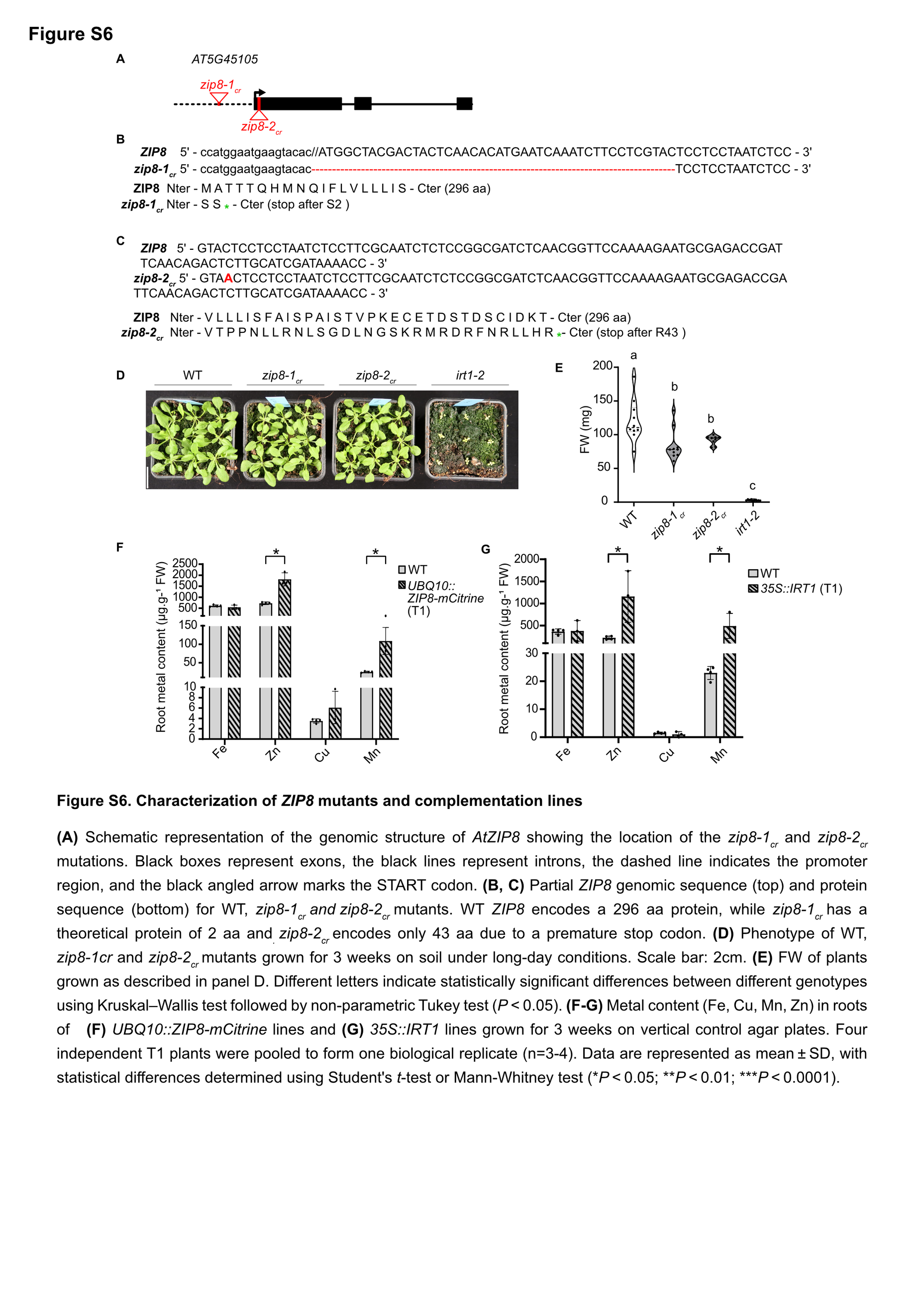

Supplement: S6 Fig — (A) Schematic representation of the genomic structure of AtZIP8 showing the location of the zip8–1cr and zip8–2cr mutations. Black boxes represent exons, the black lines represent introns, the dashed line indicates the promoter region, and the black angled arrow marks the START codon. (B, C) Partial ZIP8 genomic sequence (top) and protein sequence (bottom) for WT, zip8–1cr and zip8–2cr mutants. WT ZIP8 encodes a 296 aa protein, while zip8–1cr has a theoretical protein of 2 aa and. zip8–2cr encodes only 43 aa due to a premature stop codon. (D) Phenotype of WT, zip8–1cr and zip8–2cr mutants grown for 3 weeks on soil under long-day conditions. Scale bar: 2 cm. (E) FW of plants grown as described in panel D. Different letters indicate statistically significant differences between different genotypes using Kruskal–Wallis test followed by non-parametric Tukey test (P < 0.05). (F-G) Metal content (Fe, Cu, Mn, Zn) in roots of (F) UBQ10::ZIP8-mCitrine lines and (G) 35S::IRT1 lines grown for 3 weeks on vertical control agar plates. Four independent T1 plants were pooled to form one biological replicate (n = 3–4). Data are represented as mean ± SD, with statistical differences determined using Student’s t-test or Mann-Whitney test (*P < 0.05; **P < 0.01; ***P < 0.0001). (TIF) [file pgen.1011796.s006.tif]

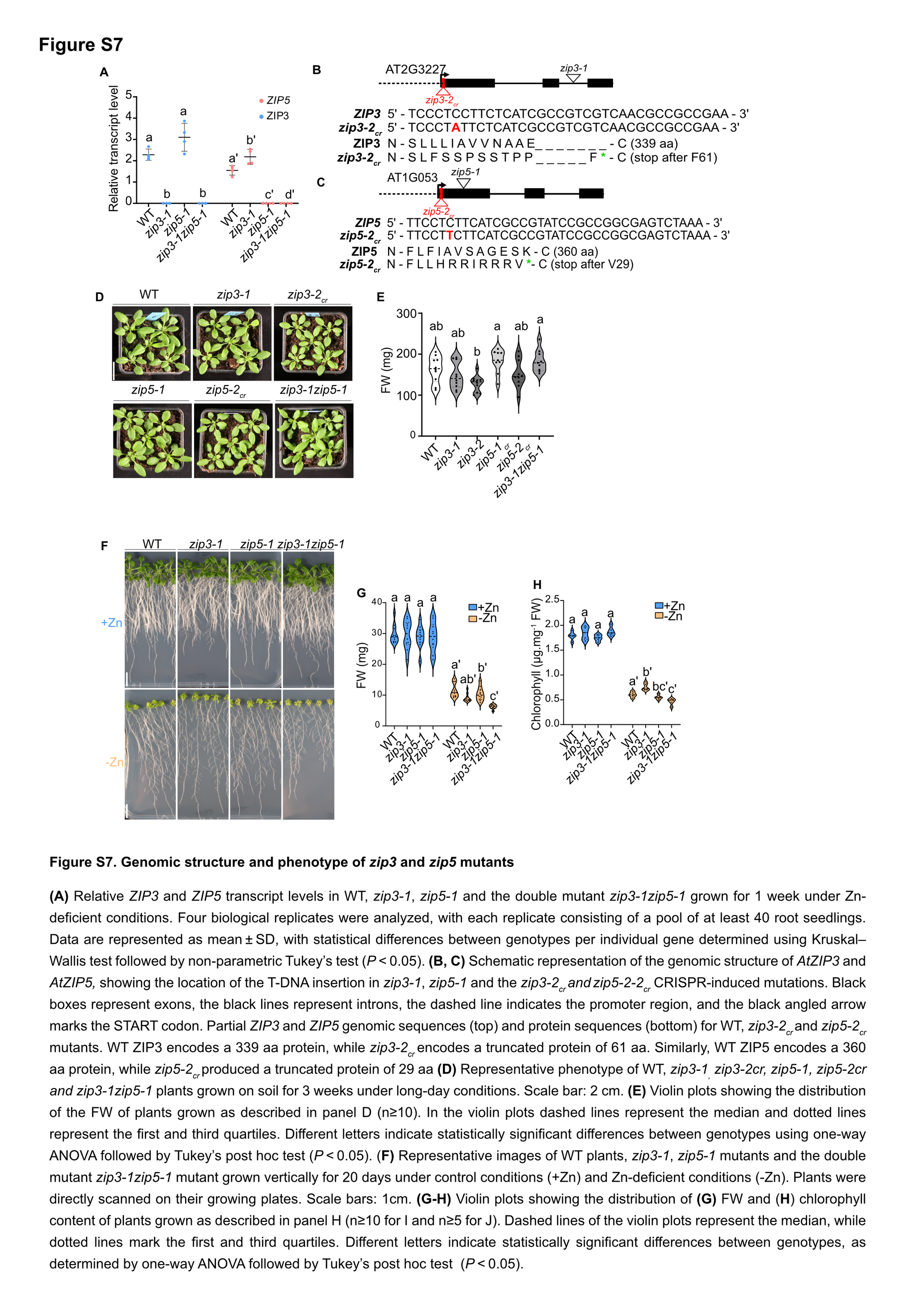

Supplement: S7 Fig — (A) Relative ZIP3 and ZIP5 transcript levels in WT, zip3–1, zip5–1 and the double mutant zip3–1zip5–1 grown for 1 week under Zn-deficient conditions. Four biological replicates were analyzed, with each replicate consisting of a pool of at least 40 root seedlings. Data are represented as mean ± SD, with statistical differences between genotypes per individual gene determined using Kruskal–Wallis test followed by non-parametric Tukey’s test (P < 0.05). (B, C) Schematic representation of the genomic structure of AtZIP3 and AtZIP5, showing the location of the T-DNA insertion in zip3–1, zip5–1 and the zip3–2cr and zip5-2-2cr CRISPR-induced mutations. Black boxes represent exons, the black lines represent introns, the dashed line indicates the promoter region, and the black angled arrow marks the START codon. Partial ZIP3 and ZIP5 genomic sequences (top) and protein sequences (bottom) for WT, zip3–2cr and zip5–2cr mutants. WT ZIP3 encodes a 339 aa protein, while zip3–2cr encodes a truncated protein of 61 aa. Similarly, WT ZIP5 encodes a 360 aa protein, while zip5–2cr produced a truncated protein of 29 aa. (D) Representative phenotype of WT, zip3–1, zip3–2cr, zip5–1, zip5–2cr and zip3–1zip5–1 plants grown on soil for 3 weeks under long-day conditions. Scale bar: 2 cm. (E) Violin plots showing the distribution of the FW of plants grown as described in panel D (n ≥ 10). In the violin plots dashed lines represent the median and dotted lines represent the first and third quartiles. Different letters indicate statistically significant differences between genotypes using one-way ANOVA followed by Tukey’s post hoc test (P < 0.05). (F) Representative images of WT plants, zip3–1,zip5–1 mutants and the double mutant zip3–1zip5–1 mutants grown vertically for 20 days under control conditions (+Zn) and Zn-deficient conditions (-Zn). Plants were directly scanned on their growing plates. Scale bars: 1 cm. (G-H) Violin plots showing the distribution of (G) FW and (H) chlorophyll content o [file pgen.1011796.s007.tif]

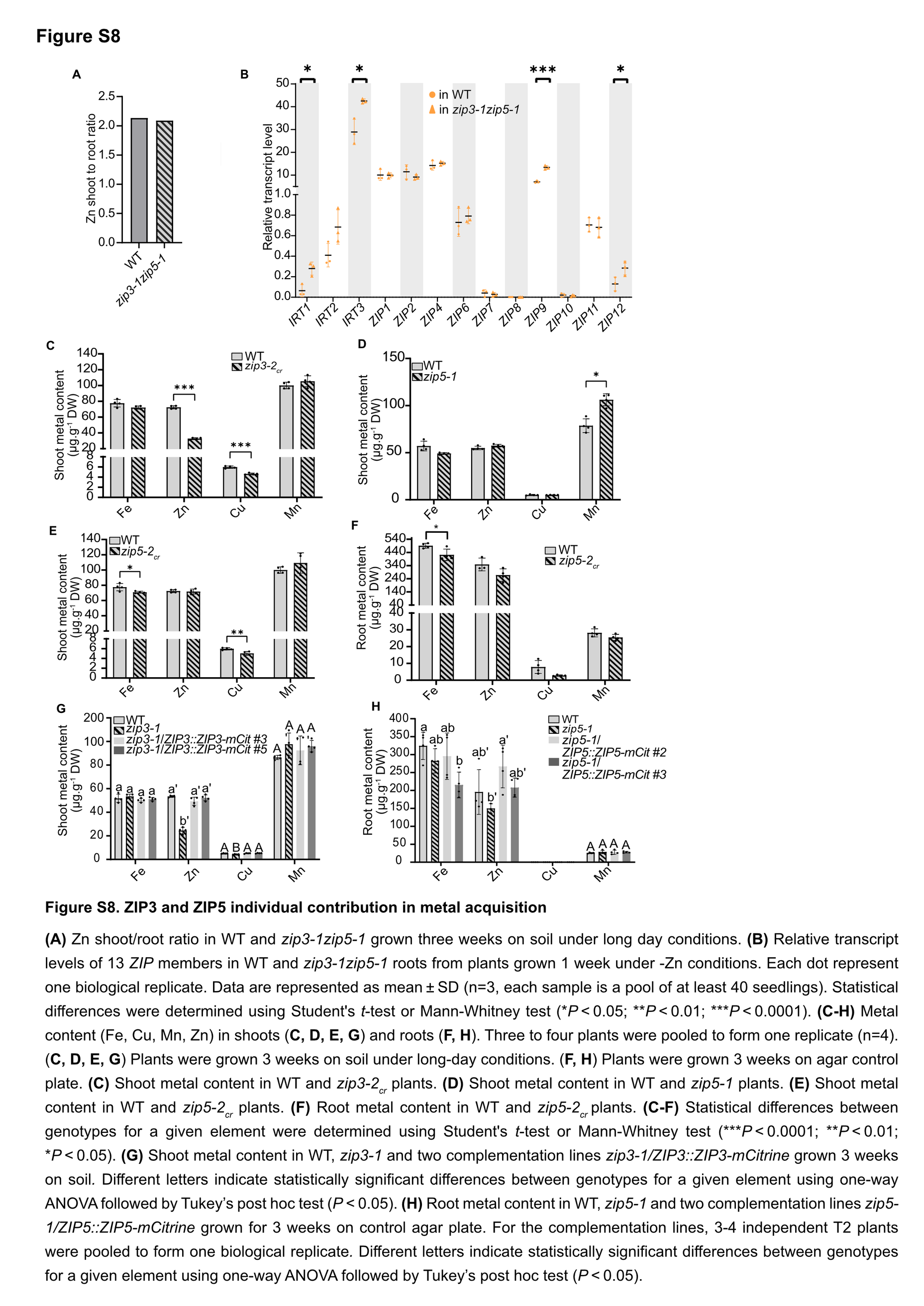

Supplement: S8 Fig — (A) Zn shoot/root ratio in WT and zip3–1zip5–1 grown three weeks on soil under long day conditions. (B) Relative transcript levels of 13 ZIP members in WT and zip3–1zip5–1 roots from plants grown 1 week under -Zn conditions. Each dot represent one biological replicate. Data are represented as mean ± SD (n = 3, each sample is a pool of at least 40 seedlings). Statistical differences were determined using Student’s t-test or Mann-Whitney test (*P < 0.05; **P < 0.01; ***P < 0.0001). (C-H) Metal content (Fe, Cu, Mn, Zn) in shoots (C, D, E, G) and roots (F, H). Three to four plants were pooled to form one replicate (n = 4). (C, D, E, G) Plants were grown 3 weeks on soil under long-day conditions. (F, H) Plants were grown 3 weeks on agar control plate. (C) Shoot metal content in WT and zip3–2cr plants. (D) Shoot metal content in WT and zip5–1 plants. (E) Shoot metal content in WT and zip5–2cr plants. (F) Root metal content in WT and zip5–2cr plants. (C-F) Statistical differences between genotypes for a given element were determined using Student’s t-test or Mann-Whitney test (***P < 0.0001; **P < 0.01; *P < 0.05). (G) Shoot metal content in WT, zip3–1 and two complementation lines zip3–1/ZIP3::ZIP3-mCitrine grown 3 weeks on soil. Different letters indicate statistically significant differences between genotypes for a given element using one-way ANOVA followed by Tukey’s post hoc test (P < 0.05). (H) Root metal content in WT, zip5–1 and two complementation lines zip5–1/ZIP5::ZIP5-mCitrine grown for 3 weeks on control agar plate. For the complementation lines, 3–4 independent T2 plants were pooled to form one biological replicate. Different letters indicate statistically significant differences between genotypes for a given element using one-way ANOVA followed by Tukey’s post hoc test (P < 0.05). (TIF) [file pgen.1011796.s008.tif]

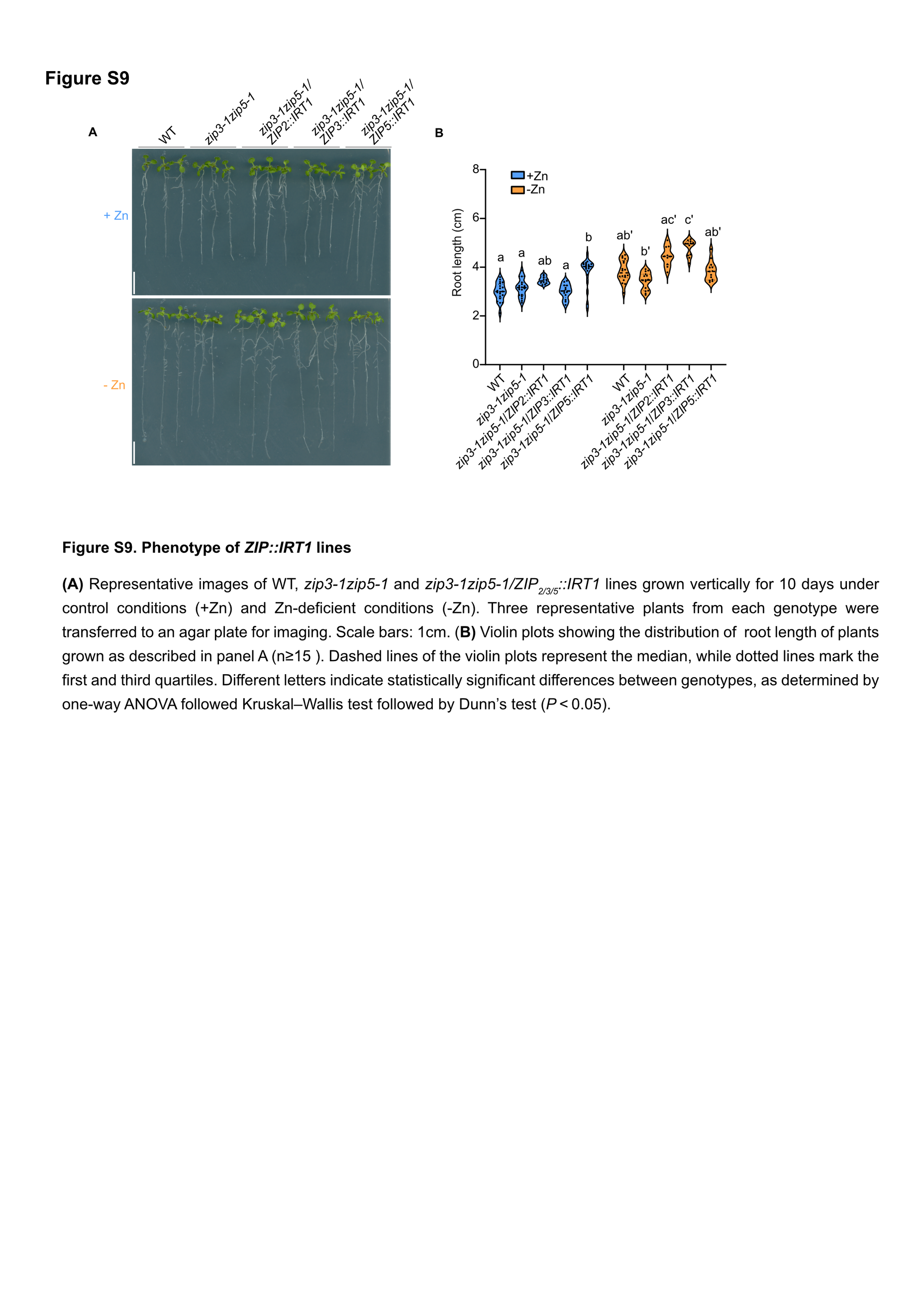

Supplement: S9 Fig — (A) Representative images of WT, zip3–1zip5–1 and zip3–1zip5–1/ZIP2/3/5::IRT1 lines grown vertically for 10 days under control conditions (+Zn) and Zn-deficient conditions (-Zn). Three representative plants from each genotype were transferred to an agar plate for imaging. Scale bars: 1 cm. (B) Violin plots showing the distribution of root length of plants grown as described in panel A (n ≥ 15). Dashed lines of the violin plots represent the median, while dotted lines mark the first and third quartiles. Different letters indicate statistically significant differences between genotypes, as determined by one-way ANOVA followed Kruskal–Wallis test followed by Dunn’s test (P < 0.05). (TIF) [file pgen.1011796.s009.tif]

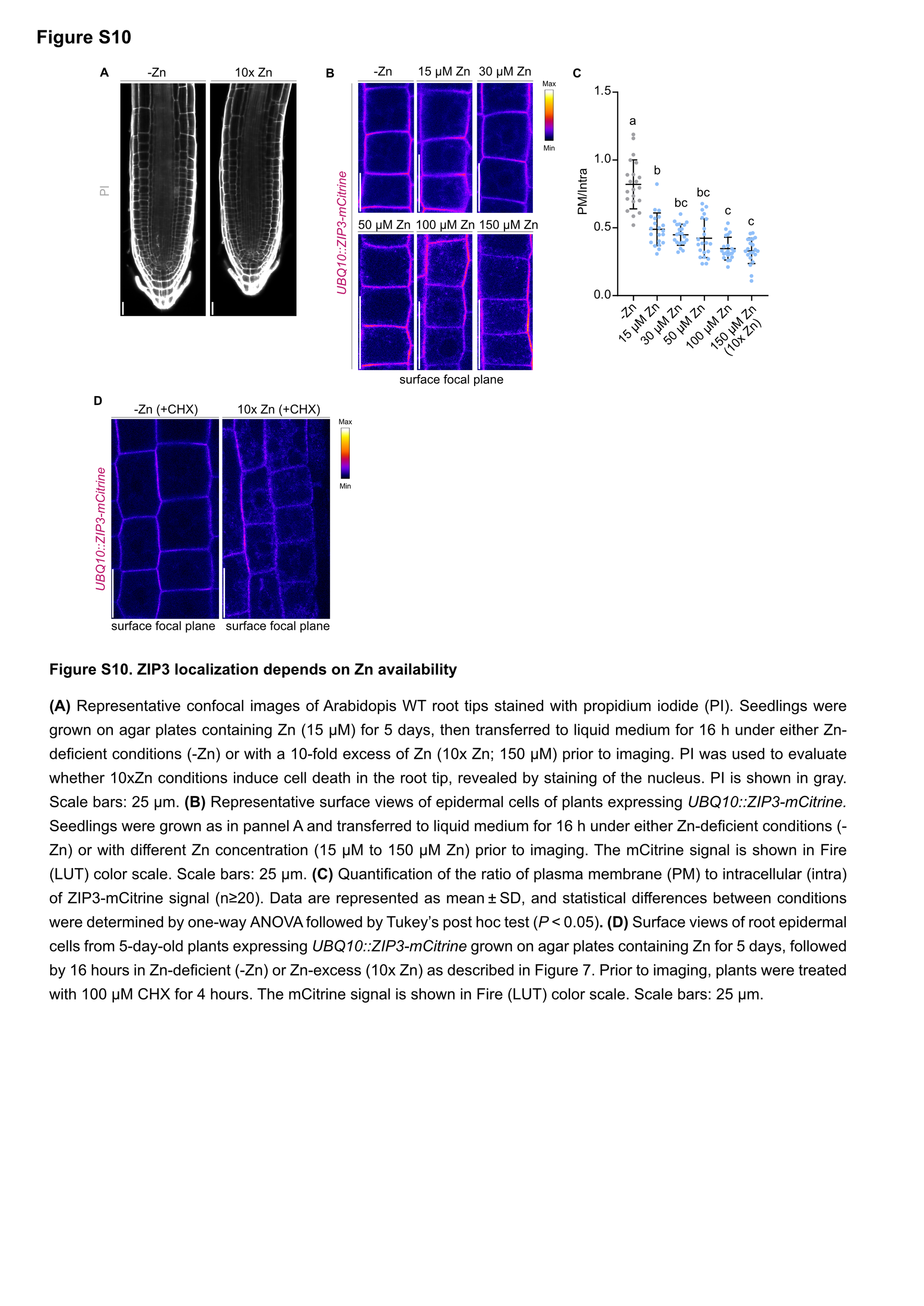

Supplement: S10 Fig — (A) Representative confocal images of Arabidopsis WT root tips stained with propidium iodide (PI). Seedlings were grown on agar plates containing Zn (15 µM) for 5 days, then transferred to liquid medium for 16 h under either Zn-deficient conditions (-Zn) or with a 10-fold excess of Zn (10x Zn; 150 µM) prior to imaging. PI was used to evaluate whether 10xZn conditions induce cell death in the root tip, revealed by staining of the nucleus. PI is shown in gray. Scale bars: 25 µm. (B) Representative surface views of epidermal cells of plants expressing UBQ10::ZIP3-mCitrine. Seedlings were grown as in pannel A and transferred to liquid medium for 16 h under either Zn-deficient conditions (-Zn) or with different Zn concentration (15 µM to 150 µM Zn) prior to imaging. The mCitrine signal is shown in Fire (LUT) color scale. Scale bars: 25 µm. (C) Quantification of the ratio of plasma membrane (PM) to intracellular (intra) of ZIP3-mCitrine signal (n ≥ 20). Data are represented as mean ± SD, and statistical differences between conditions were determined by one-way ANOVA followed by Tukey’s post hoc test (P < 0.05). (D) Surface views of root epidermal cells from 5-day-old plants expressing UBQ10::ZIP3-mCitrine grown on agar plates containing Zn for 5 days, followed by 16 hours in Zn-deficient (-Zn) or Zn-excess (10x Zn) as described in Fig 7. Prior to imaging, plants were treated with 100 µM CHX for 4 hours. The mCitrine signal is shown in Fire (LUT) color scale. Scale bars: 25 µm. (TIF) [file pgen.1011796.s010.tif]

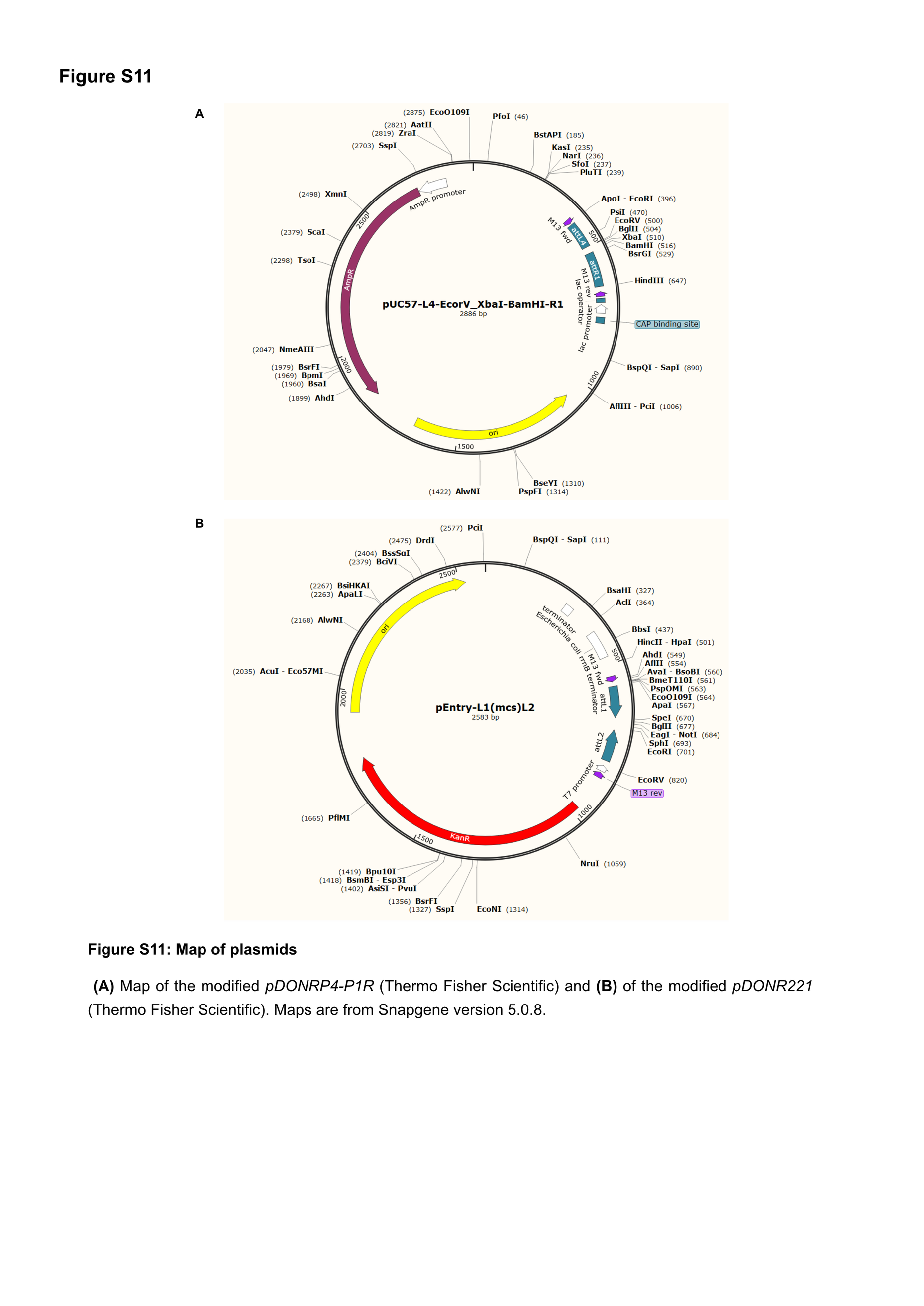

Supplement: S11 Fig — (A) Map of the modified pDONRP4-P1R (Thermo Fisher Scientific) and (B) of the modified pDONR221 (Thermo Fisher Scientific). Maps were generated with Snapgene version 5.0.8. (TIF) [file pgen.1011796.s011.tif]
