# Supplementary material for: Four ZIPs contribute to Zn, Fe, Cu and Mn acquisition at the outer root domain
Source: PLoS Genet. 2025 Jul 15;21(7):e1011796. doi: 10.1371/journal.pgen.1011796 (PMC12279101; doi:10.1371/journal.pgen.1011796)
Supplement: Attachment — Submitted filename: Robe_etal_Response_to_Reviewers final.pdf [file pgen.1011796.s018.pdf]

Dear Editors and Reviewers,

We thank you for considering our manuscript PGENETICS-D-25-00240 entitled "*Four ZIPs contribute to Zn, Fe, Cu and Mn acquisition at the outer root domain*", and for the positive feedback and constructive comments provided by all three reviewers.

We have now submitted a revised version of the manuscript in which we address all reviewers' comments, either through additional experiments or by clarifying the text as needed.

In particular, we have included new data to strengthen and expand our conclusions, including:

- *ZIP* promoter activities under Zn deficiency (Figure S2B)
- Additional evidence for ZIP8's role in Fe transport using *fet3fet4* yeast complementation assays (Figure 4A)
- Zinc uptake assays in WT and *zip3zip5* double mutants (Figure 6D)
- Long-term growth analysis of *zip3*, *zip5* and *zip3zip5* mutants on +Zn or - Zn plates (Figure S7F-H)
- Zinc content analysis in the single and double *zip3zip5* mutant grown under low Zn availability (Figure 6C, 6E)
- qRT-PCR analysis of *ZIP* gene expression in *zip3zip5* background (Figure S8B)
- Confirmation of the absence of cell death in our Zinc excess conditions (Figure S10A)
- ZIP3 localization analysis (plasma membrane vs. intracellular) across a broader range of Zn concentrations (Figure S10B).

Consequently, we had to reorganize our figures compared to the previous version of our manuscript.

We are grateful for your insightful suggestions, which have significantly improved the quality and clarity of our work.

Below, you will find our detailed point-by-point responses to the reviewer's comments.

Our responses are marked in green, and modifications to the manuscript are indicated in blue.

With our best regards.

Marie Barberon and Kevin Robe

Reviewer #1:

In this study by Robe et al., the authors functionally characterized four ZIP family transporter in Arabidopsis, in terms of expression pattern, tissue localization, metal-dependent phenotypic analysis of knockout lines. Firstly, they found that ZIP2 is a Cu transporter and potentially an Mn transporter, involved in Cu and Mn acquisition from soil. Secondly, they found ZIP8 is an Fe transporter which is polarly localized at the epidermis face to the soil side. Thirdly, they found ZIP3 and ZIP5 function redundantly in the uptake of Zn in the outer root domain, and they also observed the substrate induced endocytosis of ZIP3 in response to Zn excess. The overall presentation of the work is well done, and the study is easy to follow. To enhance the manuscript's clarity and quality, I suggest addressing the following points.

We thank Reviewer 1 for the very positive and encouraging comments on our manuscript.

We have carefully addressed the reviewer's suggestions in the revised version and have added several experiments to further improve the clarity and overall quality of the manuscript.

Please find our detailed responses below.

Major:

1. In figure 1 E, F, is it necessary to present the result of Al? Since Al is mainly active at low PH condition. What is the pH of the culture medium? Actually, I don't think figure 1 is necessary, as it is commonly Known that Zn deficiency affects the growth of plant and also mineral elements accumulation.

Response: We thank the reviewer for this helpful suggestion. We agree that including Al in the plot was not appropriate given the pH of our media. In fact, the reviewer's comment made us realize that we had not specified the pH in the Materials and Methods section. We now clarify that all plate-based experiments were conducted using media adjusted to pH 5.7. We believe that the effects of zinc deficiency are still not well-characterized, particularly in dicot plants, and that the data presented in this figure may be of general interest to a broad readership, including those less familiar with the field of metal nutrition. To our knowledge, ionomic profiling of Arabidopsis roots and shoots under +Zn and –Zn conditions has not previously been reported. To improve clarity, we have moved the former Figure 1 to the Supplemental Data as Figure S1 in the revised version of the manuscript.

Modifications:

- All references to aluminum (Al) have been removed from the manuscript.
- The pH of the media (5.7) has been added to the Materials and Methods section (line 666).
- Former Figure 1 is now Supplemental Figure S1, and we have included the root-to-shoot mineral ratio (originally in S1) in this new figure.
- The text has been adjusted accordingly (line 137-138).

2. The authors demonstrated that ZIP2, ZIP8, and ZIP3, 5 were involved in the uptake of Cu, Fe, and Zn respectively. However, they only showed the mineral element accumulation in roots and shoots. For more direct evidence in element uptake, I suggest using the stable isotopes of these elements for short-term uptake experiments.

Response: We thank the reviewer for this insightful comment. In the original version of our manuscript, we made an effort to avoid using the term "uptake" since we didn't provide direct experimental evidence. Instead, we preferred terms like "accumulation" or "acquisition" to avoid overinterpretation. However, thanks to the reviewer's careful reading, we realized that "uptake" still appeared in some parts of the Results section, particularly in relation to ZIP2 and ZIP8. We have now systematically replaced the term "uptake" with "acquisition" throughout the manuscript wherever no direct evidence of uptake is provided.

To strengthen the main conclusion of our study, we have now included short-term zinc uptake assays in WT and *zip3zip5* double mutant plants. These assays revealed a significant reduction in Zn uptake in the mutant 6 hours after Zn resupply (now shown in Figure 6D), supporting the role of ZIP3 and ZIP5 in root Zn uptake. Similar findings were also reported by Ochoa Tufiño *et al.* (2025), reinforcing our conclusions.

Regarding ZIP2 and ZIP8, we agree that direct *in planta* Fe and Cu uptake measurements would further support their proposed roles. However, due to time constraints and the need to optimize specific growth and uptake conditions (which would likely require several months of work, based on our Zn assays), we were not able to obtain these data during the revision process.

Nonetheless, we believe that the Cu transport assay reported by Wintz *et al.* (2003) already provides strong evidence for ZIP2's ability to transport Cu. Additionally, we now include new data showing that ZIP8 can partially rescue the growth defect of the *fet3fet4* yeast mutant defective in iron uptake (Figure 4A). Although ZIP8's complementation is not as robust as IRT1, this supports a minor role for ZIP8 in Fe acquisition.

Modifications:

- We added short-term Zn uptake experiments as Figure 6D.

Figure 6D

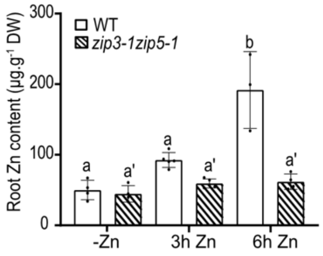

- We included a yeast complementation assay for ZIP8 as Figure 4A.

Figure 4

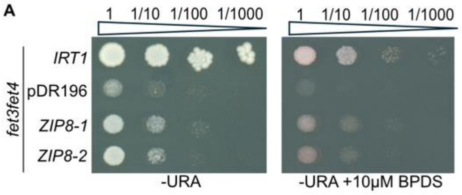

- We revised the manuscript text to describe these new results (line 300 to 308 for yeast complementation assays and line 387 to 394 for Zn uptake), and they are discussed in the Discussion section (line 516-517 and line 537-538).
- We replaced all instances of "uptake" with "acquisition" in the relevant sections where uptake was not experimentally demonstrated.

3. It appears that the expression of ZIP2, ZIP8, and ZIP3, 5 is induced by Cu, Fe, and Zn deficiency respectively, I recommend to determine the mineral element accumulation under low-concentration treatment condition, which may make the physiological phenotypes clearer.

Response: We fully agree with the reviewer that measuring metal content under lower external concentrations would be valuable and could potentially uncover differences that are masked by the relatively high metal concentrations in MS-based media. Although MS medium contains a relatively low concentration of Cu (50 nM, approximately 10 times lower than standard Hoagland medium), it does contain comparatively high levels of Zn, which could indeed skew metal homeostasis and mask phenotypic differences.

To address this concern, we analyzed Zn content in plants grown under more physiologically relevant conditions—specifically, in soil, which provides lower and more variable Zn availability. We now include data showing Zn concentrations in both roots and shoots from soil conditions of WT, *zip3-1*, *zip5-1*, the *zip3-1zip5-1* double mutant, and a newly generated CRISPR mutant line (*zip3-2crzip5-2cr*). These results further reinforce the predominant role of ZIP3 over ZIP5 in Zn acquisition (shown in Figure 6C and E).

Importantly, whether Zn is supplied at moderate or low concentrations, consistent trends are observed—most notably, a significant reduction in root Zn content in the *zip3zip5* double mutant compared to WT. Our findings are also consistent with those reported in Ochoa Tufiño *et al*. (2025), who observed a similar reduction in shoot Zn content in *zip3zip5* mutants grown on Hoagland medium, further supporting the conclusions of both studies.

Unfortunately, due to time constraints and the limited availability of the ICP-OES instrument required for ionomic analysis, we were unable to perform additional elemental profiling on plants grown on Zn-, Cu- or Fe-deficient agar plates during this revision period.

Modifications:

- We added new data showing shoot metal content in soil-grown *zip3-1zip5-1* (T-DNA mutant) and *zip3-2crzip5-2cr* (CRISPR mutant) plants (Figure 6C).

Figure 6

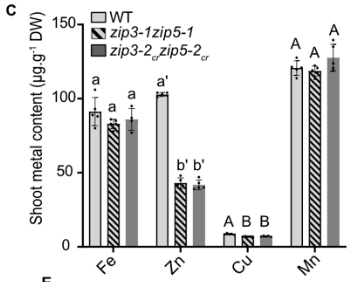

- We also provide root and shoot Zn concentrations for WT, *zip3-1*, *zip5-1*, and *zip3-1zip5-1* soil-grown plants (Figure 6E).

Figure 6

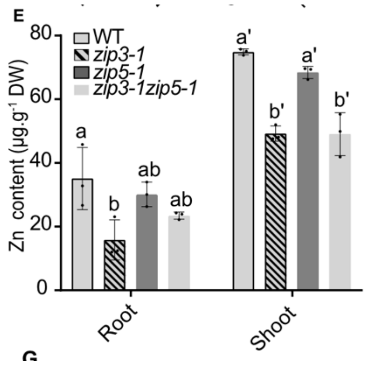

- The Results section has been revised to describe these findings (line 371-377 and 395-404), and the data are now discussed in the Discussion section (line 539-546).

4. Line 291, for +Fe condition, which form of the Fe was used? Line 298-301, if ZIP8 is involved in Fe uptake, please explain why the accumulation of Fe decreased only in the root of KO lines but not shoot. As I mentioned previously, it is better to perform a short-term uptake experiment by using a stable isotope to directly show the function in uptake.

Response: We thank the reviewer for pointing this out and apologize for the oversight. Indeed, we used Fe-EDTA as the iron source in all experiments, and we have now clarified this in the Materials and Methods section.

Regarding the apparent discrepancy between root and shoot metal levels, we agree that it may seem surprising that shoot Fe content remains unchanged while root Fe levels are affected. However, this phenomenon has been reported in several studies on mineral homeostasis. For example, Caillatte et al. (2010) showed that while the *nramp1* mutant exhibited significantly reduced Mn content in roots compare to WT, Mn levels in the shoots remained comparable to WT. It has been proposed that under certain conditions, plants may actively prioritize the translocation of essential minerals to the shoot to maintain key physiological functions. This could explain the maintained shoot Fe levels in our mutants despite lower accumulation in the roots.

Modifications:

- The Fe source (Fe-EDTA) used throughout the study is now specified in the Materials and Methods section (line 668-669).
- We have added a brief discussion of this potential root/shoot discrepancy in Fe levels in the Discussion section (line 517-521), citing Cailliatte et al., 2010.

5. For ZIP3/ZIP5, similar to ZIP8, if ZIP3 and ZIP5 is involved in Zn uptake, please explain or discuss further why Zn accumulation decreased only in root but not in the shoot of the double mutant.

Response: We agree with the reviewer that the unchanged shoot Zn content in the *zip3zip5* double mutant grown on agar plates is intriguing. As noted in our response to comment 4, this may reflect a prioritization of Zn translocation to shoots under these conditions. Alternatively, the high Zn concentration present in MS-based media may mask more subtle differences in shoot Zn levels between genotypes.

Moreover, as mentioned in the manuscript (line 351-357), reduced evapotranspiration on agar plates may limit xylem-driven translocation of Zn to the shoot, thereby obscuring phenotypes that would otherwise be more apparent in soil-grown plants. This has previously been demonstrated for boron (Muro et al., 2023). In support of this hypothesis, we observed that both *zip3* and *zip3zip5* mutants exhibit reduced shoot Zn levels when grown in soil compared to WT plants (Figure 6E), reinforcing the role of these transporters in Zn uptake and translocation.

Importantly, we have now added direct Zn uptake assays in hydroponically grown plants. These experiments reveal a strong reduction in Zn uptake in the *zip3zip5* double mutant compared to WT (Figure 6D), further confirming the essential contribution of ZIP3 and ZIP5 to Zn acquisition.

Modifications:

- We mentioned the potential influence of media Zn levels and evapotranspiration on shoot Zn content in the result section (line 371-378) and discussion section (line 542-545)
- Data from soil-grown plants showing reduced Zn in *zip3* and *zip3zip5* shoots were included (Figure 6E).

Figure 6

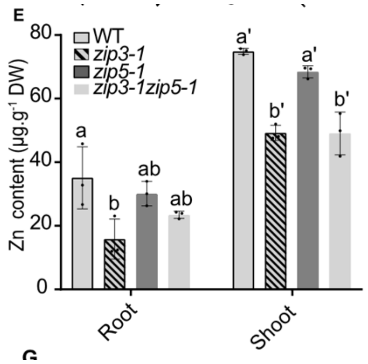

- New Zn uptake experiments were added as Figure 6D, and are described in the revised Results section (line 387-394).

Figure 6D

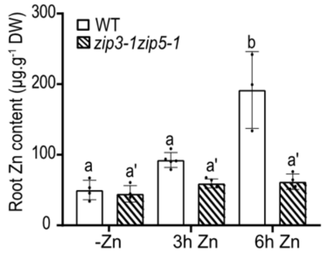

6. The authors stated that by using UBQ10::ZIP3-mCitrine plants, ZIP3 protein levels are affected by high zinc-induced endocytosis. How about under the endogenous promoter condition? Does ZIP3 synthesis itself respond to zinc concentration? If protein synthesis is inherently influenced by zinc concentration (for example, if high Zn significantly inhibits the synthesis of the protein level of ZIP3), then study on protein degradation would become less meaningful.

Response: We thank the reviewer for pointing out the lack of clarity in this section and would like to take this opportunity to clarify our rationale. The goal of the final set of experiments was to investigate post-translational effects of Zn availability on the ZIP3 protein. As shown in our study (now Figure 1B), ZIP3 is strongly regulated at the transcriptional level under Zn-deficient conditions, and we observed that ZIP3-mCitrine protein is barely detectable in +Zn conditions (Figure 2E). To circumvent transcriptional regulation and focus specifically on post-translational responses, we expressed ZIP3-mCitrine under the constitutive *UBQ10* promoter, ensuring constant transcript levels regardless of Zn status.

Furthermore, we used cycloheximide (CHX) pretreatment to block new protein synthesis, allowing us to monitor the behavior of the existing pool of ZIP3 protein in response to Zn resupply. This strategy enabled us to assess whether Zn availability induces post-translational changes, such as protein internalization.

To strengthen our conclusions, we have now included new data showing that lower Zn concentrations are also sufficient to trigger ZIP3 internalization (Figure S10B, S10C).

Additionally, we demonstrate that the high Zn concentrations used in our assays do not induce cell death at the root tip (Figure S10A), ruling out toxicity as a confounding factor.

Modifications:

- We clarified in the text the rational of using the *UBQ10* promoter to study post-translational regulation (line 444-446).
- We revised the text to clearly distinguish between transcriptional and post-translational regulation (line 452, 473-4574, 482).
- We added new data demonstrating that the high Zn concentrations used do not trigger cell death (Figure S10A) and that lower Zn levels also induce ZIP3 internalization (Figure S10B, S10C).

Figure S10

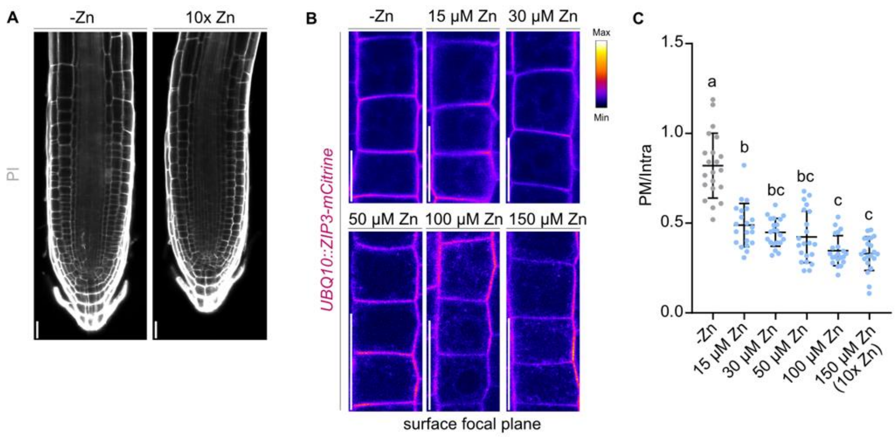

Minor:
1. Figure 4 E and F, it would be better to show results from two independent knockout lines here.

Response: We fully agree with the reviewer's comment and have modified the manuscript accordingly to strengthen the conclusions regarding ZIP2. We now provide data from two independent mutant alleles in the same figure, allowing for a more robust interpretation of the phenotype.

Modifications: We now include both shoot and root Zn content for WT, *zip2-1* (T-DNA insertion line), and *zip2-2cr* (CRISPR mutant) in the same figure (now Figure 3E and 3F). We removed the previous experiment showing only *zip2-1* mutant metal content, but data on *zip2-1* metal content remain available in Figure S5I (previously Figure S4J). The data are now presented in the Result section (line 288-290).

Figure 3

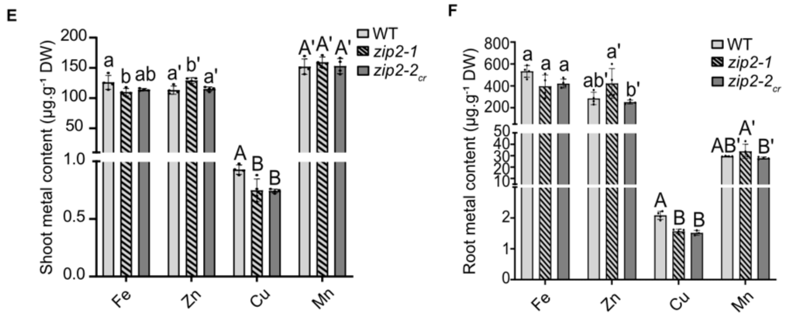

2. In figure 5 E, why are there two "WT" presented? But in figure 5F, there is only one WT.

Response: We agree with the reviewer that Figure 5E could be confusing to readers. This point was also raised by Reviewer 2. To clarify, the data shown in Figure 5E were obtained from two independent experiments, while the data in Figure 5F were generated from a single experiment. We acknowledge that this may not have been sufficiently clear in the original figure layout.

Modifications: We have revised the current version of Figure 4F (formerly Figure 5F) to better distinguish between WT, *zip8-cr1*, and *zip8-cr2*, including increased spacing and clearer labeling. We also clarified this point in the figure legend to help readers interpret the data more easily.

Figure 4

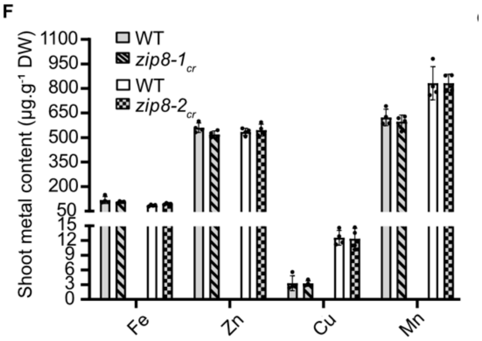

3. Line 470-473, if ZIP3 and ZIP5 are not involved in root-to-shoot translocation, may be check the expression of other ZIPs in zip3/zip5 double mutants.

Response: This is indeed an interesting point. To explore whether other ZIP transporters might contribute to root-to-shoot Zn translocation in the absence of *ZIP3* and *ZIP5*, we performed qPCR analysis on roots of WT and *zip3zip5* mutants grown under Zn-deficient conditions. Notably, we observed increased transcript levels of *IRT1*, *IRT3*, *ZIP9*, and *ZIP12* in the *zip3zip5* mutant compared to WT. This suggests that these ZIPs may partially compensate for the loss of *ZIP3* and *ZIP5* and could potentially be involved in Zn

translocation to the shoot under deficiency conditions. However, we agree that further in-depth studies would be required to confirm such a role, and we have remained cautious in our interpretation. Interestingly, a similar increase in *IRT3* expression was also reported by Ochoa Tufiño et al., 2025, further supporting our observations.

Modifications: We added a new supplementary figure (Figure S8B) presenting the qPCR data on ZIP transcript abundance in roots of WT and *zip3zip5* mutants. We also briefly discuss the possible implications of this differential expression in the result section (line 379-386).

Figure S8

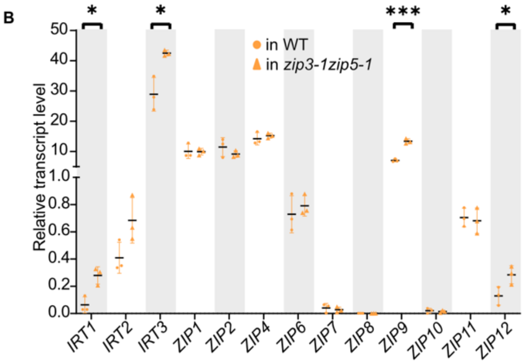

Reviewer #2:

The authors provide various types of data for functionally characterizing genes encoding proteins of the ZRT-IRT-related protein family that are candidates for roles in micronutrient metal uptake. The data are partly confirmatory (e.g. contribution of ZIP2 in Cu uptake; Wintz et al., 2003, and others) but add some new or additional data compared to previous publications. All in all, however, the manuscript provides some interesting observations, while the reasoning for narrowing down to specific transporters and some of the conditions used, ..., are somewhat incomplete, sketchy, suboptimal or limited (e.g., protein localization data are only shown for a small set of ZIP proteins and conditions, Fig. 3). The authors should alert readers in the results to the limitations and also discuss and their implications specifically (see below). The suggested contributions of ZIP3 and ZIP5 to Zn uptake supported by the data in this manuscript have recently been published by others (10.1111/tpj.17251), yet additional support remains of scientific value at this point. A (minor, but biologically relevant) contribution of ZIP8 to Fe uptake is novel and was not previously shown.

We thank Reviewer 2 for her positive and encouraging evaluation of our manuscript. We are especially grateful for the thorough and thoughtful analysis, which helped us identify and correct several inaccuracies and oversights.

In response to her comments, we have carefully revised the manuscript, added several new experiments, and clarified the presentation and discussion of key results to improve the clarity and overall quality of the work.

Please find our detailed point-by-point responses below.

Major points

1. Line 140-143 and associated data/Figures: There is an issue with the authors' data on ZIP transcript levels upregulated in Zn-deficient plants: It is widely known and published by numerous authors that ZIP4 transcript levels (and its promoter activity, based on pGUS lines) are upregulated under Zn deficiency and that ZIP6 transcript levels aren't. The authors should check their primer sequences and data, and/or repeat their experiments (mixup?). If the data from the authors remain contradictory to all previous publications, they should provide an explanation for this in the discussion, or at least explain the limitations of their data.

> **<u>Response:</u>** We thank the reviewer for this valuable comment. Indeed, this was a mistake on our part — the primers for *ZIP4* and *ZIP6* were inadvertently inverted. We had followed the primer descriptions from Lee et al. (Plant Journal, 2021) without independently verifying their identity, which explains why *ZIP4* expression did not appear to be induced by Zn deficiency, while *ZIP6* was. We have now corrected this error.
>
> **<u>Modifications</u>** : The correction has been implemented in Figure 1B, Figure S2A, and the new Figure S8B.

Figure 1

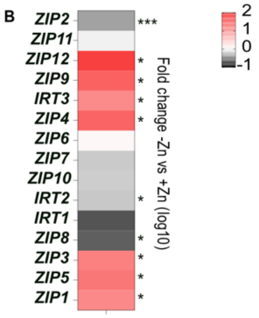

Figure S8

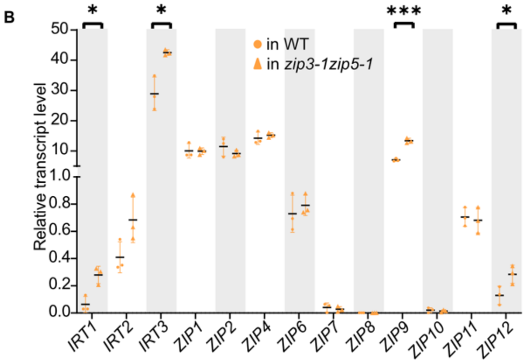

Figure S2

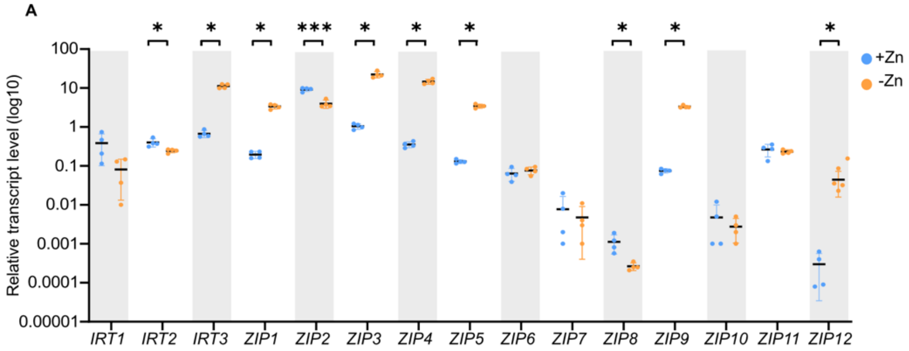

2. Line 146 and following: The argument that higher transcript levels of ZIP2 than of other ZIPs under control conditions indicate a potential role in basal metal uptake is not valid in this form. Even lower transcript levels of other ZIPs are compatible with such roles, and it is clear that there is substantial regulation at the protein level in this family (and this manuscript supports this). The authors should modify the text accordingly.

Response: We fully agree with the reviewer's comment and appreciate the suggestion to refine our interpretation.

Modifications: We have removed all mentions of a role for ZIP2 in basal metal uptake throughout the manuscript to avoid overstatements and ensure a more accurate presentation of our findings.

3. Lines 148 to 171: The reviewer sees it as a major limitation of this manuscript that images showing detection and localization of promoter activities for ZIPs are only shown for control conditions (Fig. 2C) and not for Zn deficiency conditions, because growth conditions often result in changes of the extent and domains of promoter activities. Although they later show information for specific deficiency conditions for various individual selected ZIPs, they use the limited data presented in Fig. 2C to select candidates for uptake functions. The authors should therefore highlight the limitations of this approach both here in the results (adjust the reasoning provided to narrow down among ZIPs in this section) and also again in the discussion. The reviewer also feels that this part of the text over-interprets the data shown, and thus should be changed. The authors should also clarify in the caption text of Fig. 2C that seedlings were cultivated in control conditions in order to clarify this.

Response: We fully agree with the reviewer's assessment that our initial screening approach is biased, as growing seedlings in the presence of Zn can influence the identification of candidate genes. However, our aim was to prioritize ZIP transporters likely to contribute to metal acquisition under a broader range of conditions, not solely during deficiency. Although biased, this strategy successfully led us to uncover previously uncharacterized functions for several ZIPs—some of which were independently supported by recent findings from Ochoa Tufiño et al., 2025.

That said, we fully acknowledge that other ZIPs likely play important roles in Zn uptake, particularly under deficiency. To address this, we now include new data on the promoter activity of several Zn deficiency–responsive *ZIPs* (*ZIP1*, *ZIP3*, *ZIP4*, *ZIP5*, *ZIP9*, *ZIP12*, and *IRT3*) under low-Zn conditions. We have also clarified the growth conditions used for our *promoter::nls-3xmVenus* studies in the figure legend to avoid potential confusion.

Modifications: We added new promoter activity data under Zn-deficient conditions as Figure S2B. We now discuss the limitations of our initial approach and the likely involvement of additional ZIPs in Zn uptake in both the results and discussion sections (lines 185-194, 557-564). We also briefly compare our findings to those of Ochoa Tufiño et al., 2025 (line 191-192, 536).

Figure S2

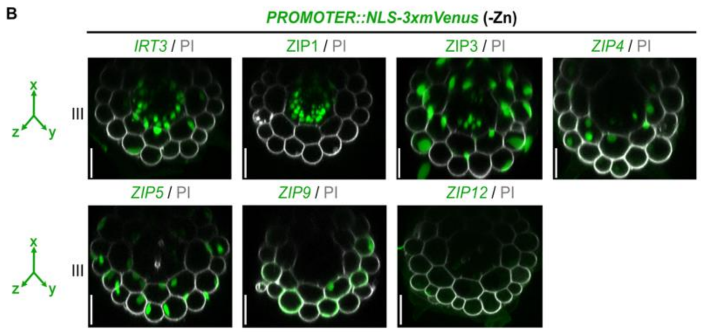

4. In this context, the conclusion for ZIP4, ZIP9 and ZIP12 promoter activity was minimal (line 161) is not supported by the shown images (Fig. 2C) in the present form, and the fact that these images are from control conditions is decisive and should be mentioned here also, at least. Also the statement in line 166-167 has the limitation of referring to control conditions only. A naive reader would observe activity in epidermal cells also for ZIP4, ZIP9 and ZIP12 (Fig. 2C). The authors don't argue or provide evidence for disregarding this despite some green signal shown in the epidermal and/or cortex cell layers in Fig. 2C (please consider also the results shown here 10.1111/tpj.17251). Transcript levels in Fig. S1 are consistent with a major role for ZIP9 under zinc deficiency (ZIP4 data are questionable – see above), and also ZIP12 cannot be entirely discounted as its transcript levels are strongly upregulated under Zn deficiency.

Response: We agree that ZIP4, ZIP9, and ZIP12 are strong candidates for playing a major role under zinc-deficient conditions, and we acknowledge that our screening approach, which was conducted under zinc-sufficient conditions, may have limited our ability to uncover such roles. As mentioned in our previous response, we have now examined the promoter activity of these ZIPs under Zn-deficient conditions (Figure S2B). Notably, *ZIP4* and *ZIP9* showed strong promoter activity in the epidermis and cortex under Zn deficiency, while *ZIP12* still did not display detectable activity in our hands.

The faint, diffuse green signal observed for *ZIP12* under both Zn conditions—and for *ZIP4* and *ZIP9* under Zn-sufficient conditions—likely corresponds to background fluorescence, not promoter-driven mVenus expression. This background signal appeared only when we substantially increased the gain to ensure no weak nuclear signal was missed. We now clarify this point in the figure legend.

Since *ZIP4*, *ZIP9*, and *ZIP12* did not show detectable promoter activity under Zn-sufficient conditions, we initially focused on *ZIP2*, *ZIP3*, *ZIP5*, and *ZIP8*, which were already active in control conditions. However, we fully agree that ZIP4 and ZIP9 represent compelling candidates for future studies on zinc deficiency responses. We have also clarified in the figures and figure legends whether plants were grown in +Zn or –Zn conditions and compared our findings to those of Ochoa Tufiño et al., 2025, highlighting both similarities and differences.

Modification: We added Figure S2B showing promoter activity of *ZIP4*, *ZIP9*, and *ZIP12* under Zn-deficient conditions. We clarified in the main figures and figure legends the Zn condition used (+Zn or –Zn), explained that the faint green signal observed under some conditions represents background fluorescence (not nuclear signal or promoter activity, line 176-177), and justified our focus on ZIP2, ZIP3, ZIP5, and ZIP8. (line 195-197). We also acknowledge that others ZIP may certainly contribute to Zn uptake such as ZIP4, ZIP9 or IRT3 (line 557-564).

Figure S2

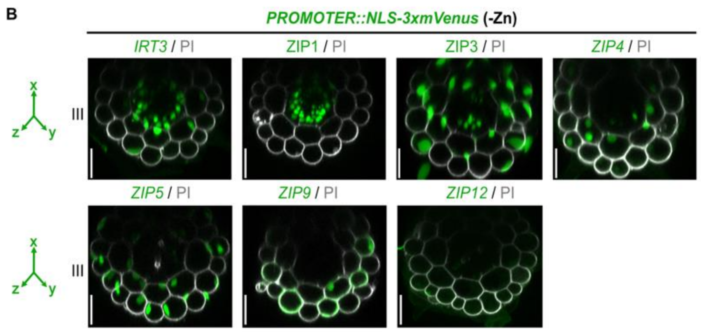

5. The reviewer sees the ionome data as another major limitation of this study. First, it was only conducted under one condition and not additionally under a controlled deficiency condition on synthetic media (e.g. Fig. 4E, Fig. 5E, Fig. 6E). Second, the numbers are strange, and this is probably a consequence of using a strangely composed medium. For example, all Cu concentrations in plant tissues are strongly in the deficient range (8 µg g-1 are sufficient, <= 4 deficient; here concentrations are at 1-2). The used media (MS-based) are well-known to be Cu-deficient (e.g. Bernal et al., 2012) and extensively discussed in the literature as highly problematic for metal nutrition research for a variety of reasons. Shoot Zn and Mn levels are very high (10- to 20-fold above normal sufficient levels) and far above nutritional needs in the luxury range, in the control medium. The interpretation of the observations should be adjusted to this explicitly, and this also indicates that all the

data of this study were obtained from plants cultivated under conditions of strongly skewed metal homeostasis. Now, some central differences between mutant and the wild type are confirmed in soil, but the authors should generally be very cautious about excluding anything based on their results. Finally, the authors apparently grew plants at high plant density on agar-based media in constant light for as long as 3 weeks. By that time, the plants will be very large, interfering with one another, and they may also have exhausted the nutrients present in the medium of their respective petri plate. So, all in all, these data are not particularly high quality. The authors should mention and discuss all these limitations. They should also add in the methods the information of how many plants were grown per plate, etc. (see also below).

Response: We thank the reviewer for this detailed and thoughtful critique of our ionomic analysis. We fully agree that this part of the study benefits from a more explicit discussion of its limitations. As correctly pointed out, the ionomic data were primarily generated under a single growth condition using MS-based medium, which has well-known compositional biases, particularly for micronutrients such as Zn. We acknowledge that additional experiments under defined nutrient-deficient conditions—especially for zinc—would have further strengthened our conclusions.

That said, several of the key genotype-dependent differences observed under MS-based conditions were also confirmed in our soil-grown plants, reinforcing the biological relevance of our findings. Furthermore, these observations are consistent with recent results reported by Ochoa Tufiño et al., 2025. We now explicitly discuss the limitations associated with our growth conditions and clearly state that the ionomic data should be interpreted in the context of this specific experimental setup.

In response to the reviewer's concerns about plant density and prolonged growth on agar, we have added more precise information in the Materials and Methods section regarding the number of seedlings per plate and the overall culture conditions. While we recognize that extended growth on agar may lead to nutrient depletion and competition, we did not observe signs of nutrient exhaustion—such as chlorosis or reduced biomass—within the timeframe of our experiments. Nonetheless, we now clearly acknowledge this as a potential limitation in the Discussion section and discuss how it may influence ionomic readouts.

Modification: We added new ionomic data from soil-grown plants, including results for the *zip3zip5* double mutant, the *zip3-1zip5-1* allele, and a new CRISPR allele (*zip3-2cr zip5-2cr*) (Figure 6C). The results section was updated accordingly (line 371-377, 395-404), and we now discuss the limitations of our ionomic analysis more explicitly in the results and Discussion (371-373, 542-545).

6. Please clarify in the manuscript explicitly which of the mCitrine fusions complement the respective mutants or double mutants (i.e., with data provided in the manuscript), which ones don't (provide the data if it exists), and for which ones this was not investigated yet. Please discuss this specifically and exhaustively. A supplemental table listing summarizing this might be helpful.

Response: We thank the reviewer for this helpful comment. By applying the strategy previously described for IRT1—namely, inserting the mCitrine tag into the predicted extracellular loop—we successfully generated functional fusions for all ZIP proteins analyzed in detail in this study (ZIP2, ZIP3, ZIP5, and ZIP8). For these four transporters, we did not encounter any non-functional fusion constructs.

In the original manuscript, we demonstrated the functionality of these tagged proteins either through complementation of their respective loss-of-function mutants (ZIP2, ZIP3, and ZIP5; Figures S5I and S8G,H) or by showing their impact on metal accumulation when overexpressed (ZIP8; Figure S6F). To improve clarity and provide a comprehensive overview, we now include a new supplementary table summarizing the functionality of each mCitrine-tagged ZIP construct.

Modification: We added Supplementary Table S2, which lists all mCitrine fusion constructs generated in this study, summarizes how their functionality was assessed, and refers to the relevant figures (line 217, 331-332).

Table S2: Functional characterization of ZIP fusion proteins

Construct	Background	Functionality	Figure
ZIP2::ZIP2-mCitrine	*zip2-1*	Yes (Restore Cu content in roots)	Figure S5I
ZIP3::ZIP3-mCitrine	*zip3-1*	Yes (Restore Zn content in shoots)	Figure S8G
ZIP5::ZIP5-mCitrine	*zip5-1*	Yes (Increase Zn content in roots)	Figure S8H
UBQ10::ZIP8-mCitrine	WT (Col-0)	Yes (Increase Zn and Mn content in roots)	Figure S6F

7. Please clarify in results and discussion why we see little visible difference between WT and zip3 zip5 in Fig. 6A (0 µM Zn), whereas 6D suggests an about 50% difference in chlorophyll level.

Response: We thank the reviewer for this comment. We agree that the phenotypic differences between WT and the *zip3zip5* mutant are not immediately striking in the original images. Although the visual difference is relatively mild, *zip3zip5* plants consistently appear less green than WT under -Zn conditions. As is often the case, capturing subtle variations in leaf coloration through photographs can be challenging. This is why we also quantified chlorophyll content, which revealed an approximately 40% reduction in the mutant.

To better illustrate the phenotype, we now provide close-up images of the shoots (Figure 5B), which more clearly show the reduced size and paler coloration of *zip3zip5* compared to WT. Additionally, we added images of 20-day-old WT, *zip3*, *zip5*, and *zip3zip5* plants grown under +Zn and -Zn conditions (Figure S7H–J). These images confirm the same phenotypic trend, without revealing differences in the single mutants.

Modification: We added close-up images of WT and *zip3zip5* seedlings grown for 10 days on plates (Figure 5B), as well as images of 20-day-old plants exposed to Zn deficiency for 10 additional days (Figure S7F–H). The corresponding text in the Results section was revised accordingly (line 360-366).

Figure 5

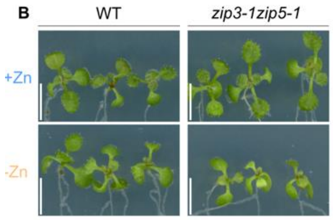

Figure S7

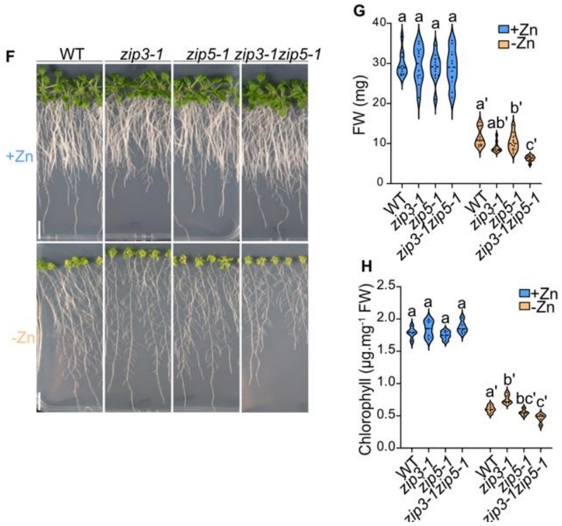

8. Please clarify in the results and discussion your interpretation of the Zn concentration data shown in Fig. 6E-H: Zn levels are far in the luxury range even in the double mutant. This comment is an example of a specific comment relating to the more general comment above (no. 5).

Response: We fully agree with the reviewer that some of our results could be the result of our specific growth conditions and that some differences could be masked by the relatively high metal concentrations in MS-based media. Although MS medium contains a relatively low concentration of Cu (50 nM, approximately 10 times lower than standard Hoagland medium), it does contain comparatively high levels of Zn, which could indeed skew metal homeostasis and mask phenotypic differences.

To address this concern, we analyzed Zn content in plants grown under more physiologically relevant conditions—specifically, in soil, which provides lower and more variable Zn availability. We now include data showing Zn concentrations in both roots and shoots from soil conditions of WT, *zip3-1*, *zip5-1*, the *zip3-1zip5-1* double mutant, and a newly generated CRISPR mutant line (*zip3-2crzip5-2cr*). These results further reinforce the predominant role of ZIP3 over ZIP5 in Zn acquisition (shown in Figure 6C and E).

Importantly, whether Zn is supplied at moderate or low concentrations, consistent trends are observed—most notably, a significant reduction in root Zn content in the *zip3zip5* double mutant compared to WT. Our findings are also consistent with those reported in Ochoa Tufiño *et al*. (2025), who observed a similar reduction in shoot Zn content in *zip3zip5* mutants grown on Hoagland medium, further supporting the conclusions of both studies.

Unfortunately, due to time constraints and the limited availability of the ICP-OES instrument required for ionomic analysis, we were unable to perform additional elemental profiling on plants grown on Zn-, Cu- or Fe-deficient agar plates during this revision period.

Modifications:

- We clarified in the text on multiple occurrence that our plates experiment were performed on rich MS-based media (lines 167-168, 176, 181, 196, 287, 319, 368).
- We added new data showing shoot metal content in soil-grown *zip3-1zip5-1* (T-DNA mutant) and *zip3-2crzip5-2cr* (CRISPR mutant) plants (Figure 6C).

Figure 6

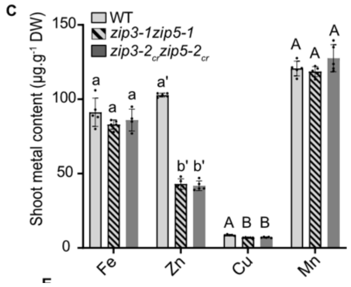

- We also provide root and shoot Zn concentrations for WT, *zip3-1*, *zip5-1*, and *zip3-1zip5-1* soil-grown plants (Figure 6E).

Figure 6

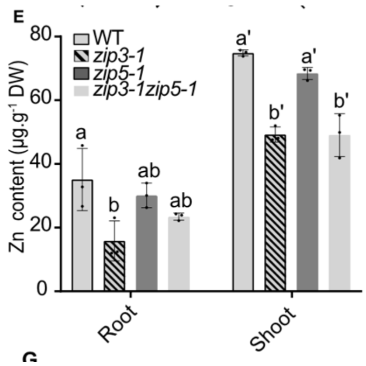

- The Results section has been revised to describe these findings (line 371-377, 395-404), and the data are now discussed in the Discussion section (line 537-538).

9. Reviewer suggests to include rice ZIP proteins in the tree in Fig. 2 (or an additional one shown in the supplement), pointing out the ones implicated in Zn uptake.

Response: We fully agree with the reviewer that a phylogenetic tree could provide useful context for readers. However, a comprehensive phylogenetic analysis of the Arabidopsis ZIP family has already been published by Zheng et al., 2018 (https://www.ncbi.nlm.nih.gov/pmc/articles/PMC6156699/). Given that this information is readily available and considering the already substantial length and number of figures in our manuscript, we feel that including an additional phylogenetic figure would not significantly enhance the manuscript and may detract from its focus.

Modification: We now cite Zheng et al. (2018) in the discussion section (line 527-528), and have now highlighted that AtZIP1 and AtZIP12 are closer to OsZIP9 and OsZIP5 than AtZIP3 or AtZIP5 are (line 528).

Minor points

10. Abstract: Suggest to delete „key“ in line 16 (explained elsewhere). Suggest to replace „underscoring the significance“ (line 19) by „in agreement with expectations“. It is also important to clarify in the abstract that statements in line 14 are limited to the conditions employed and the specific line of investigation pursued in this manuscript. Add „plants“ at the end of line 10. Reviewer feels that line 11/12 family name should be in Roman (not italic) fonts.

Response: We agree with the reviewer and have corrected the text accordingly

11. The authors should add a discussion of the somewhat surprising finding that Zinpyr fluorescence (and differences between the wild type and the zip3 zip5 double mutant) were detected only in stele and endodermis (line 354, Fig. 6F and G). The authors report the proteins to be localized in the epidermis primarily, by contrast.

Response: We fully agree that this may seem surprising at first, but we believe it reflects the specific cell types where zinc is transiently stored in the root before being translocated to the shoot. Therefore, in the *zip3zip5* double mutant—defective in transporters acting at the root periphery—it is expected that only the intensity of the signal, but not its spatial distribution, would be altered. Interestingly, a similar pattern was observed in WT roots by Ochoa Tufiño et al. (2025), although no Zinpyr-1 data were shown for the double mutant in their study.

Modifications: We now include this interpretation in the Discussion section and added a corresponding passage on lines 572–575.

12. It would be nice to see results similar to Fig. 7 also for lower Zn concentrations for readers to receive an idea of the conditions under which this is triggered – 150 µM Zn might be toxic in their medium (maybe the authors could add some marker data to examine this).

Response: We fully agree with the reviewer that verifying the absence of toxicity is essential to ensure that the observed ZIP3 relocalization is not an artifact. To address this, we performed propidium iodide (PI) staining, a standard marker for cell death, on roots exposed to 150 µM Zn (10×Zn) for 16 hours—the conditions used in our confocal experiments. In both –Zn and 10×Zn conditions, we did not observe any nuclear PI signal in the root tip, indicating that these treatments do not induce detectable cell death in this region. These results support the conclusion that ZIP3 relocalization is not a consequence of stress-induced cell death.

Additionally, we tested lower Zn concentrations on plants expressing *UBQ10::ZIP3-mCitrine* to determine the threshold required for ZIP3 delocalization. While lower Zn concentrations did trigger relocalization, a relatively high Zn supply was necessary to reproduce the extent of relocalization seen with 150 µM Zn.

Modifications: We have added new data to the revised manuscript: Figure S10A shows PI staining in roots treated with 10×Zn to assess cell viability; Figures S10B and S10C present ZIP3 localization under a range of Zn concentrations. These findings are described in the Results sections (lines 442-444, 465-468).

Figure S10

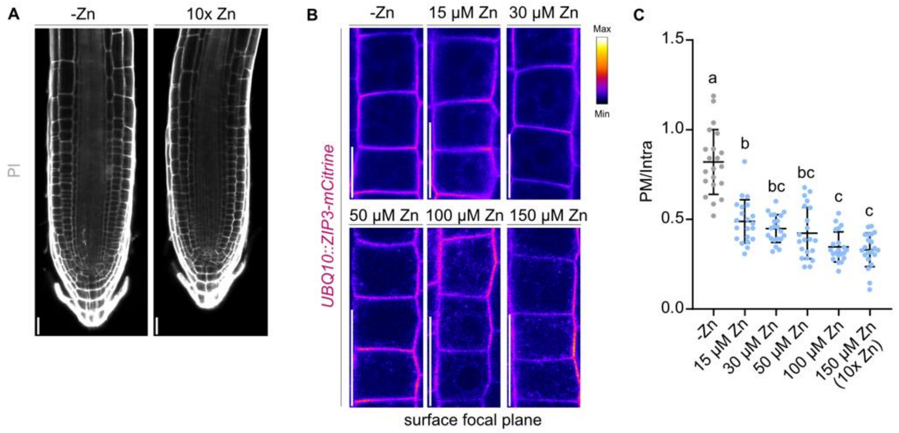

13. Line 47: cite also Van der Zaal et al. (1999) on ZAT = MTP1.

Modifications: We have made the corresponding changes in the revised manuscript (line 62).

14. Line 53: „sativa“

Modifications: We have made the corresponding changes in the revised manuscript (line 70).

15. Line 92: suggest to replace „primarily involved“ by „contribute“, given the previously published importance of CuII reduction for Cu uptake (emphasizing the roles of COPT proteins) and the major role of IRT1 in Fe uptake**.**

Modifications: We have made the corresponding changes in the revised manuscript (line 107).

16. Line 96: replace „emphasize“ by „provides circumstantial support for“ (do this also elsewhere where this argument is repeated)

Modifications: We have made the corresponding changes in the revised manuscript (line 111).

17. Line 205: „endodermal compartments“: Is this a typo?

Response: Thank you for pointing this out — it was indeed a typo.

Modifications: We have corrected it to “endosomal” in the revised manuscript (line 229).

18. Line 328: Niehs et al. has not been reviewed. Please refer to at least one peer-reviewed publication where this is shown (e.g. Sinclair et al., 2018).

Modifications: As suggested, we have now cited Sinclair et al., 2018 as a peer-reviewed publication in the revised manuscript (line 358).

19. Line 328 „significantly smaller“: All effects described should be significant. It would be far more informative for the readers if the authors provided a quantitative summary of the degree of reduction in growth.

Response: We agree with the reviewer’s comment and thank her for the suggestion.

Modifications: We have revised the text accordingly and now describe the reductions in fresh weight and chlorophyll content as percentages relative to the WT (line 359).

20. Lines 331, 360 „redundantly“: Reviewer suggests the replace by „partially redundantly“. Reviewer believes that the data provided in this manuscript rather suggest compensatory effects in mutants, and for now we do not know much about whether there is redundancy among ZIP3 and ZIP5 in wild-type plants.

Response: We agree with the reviewer’s suggestion to use “partially redundantly” instead of “redundantly.” While Ochoa Tufiño et al. (2025) describe ZIP3 and ZIP5 as functionally

redundant, our data show a clear reduction in Zn accumulation in the zip3 single mutant, but not in zip5, strongly indicating that ZIP3 and ZIP5 act only partially redundantly, at least with respect to Zn accumulation.

Modifications: We have revised the wording in the manuscript accordingly (line 419, 530-531) and added a comparison with the findings of Ochoa Tufiño et al. (2025) (line 531-542)

21. Line 431-433: Remove „we identified“ – other authors have published quite a bit of data on that. The authors provide reverse genetic evidence for this here.

Modifications: We have made the corresponding changes in the revised manuscript (line 498).

22. Line 339: The authors should specify the nutrients addressed in this cited study and also mention the well-established knowledge on the central importance of root-pressure/xylem loading dependent flux in the xylem.

Modifications: We have now specified that this phenomenon has been particularly well demonstrated for boron (Muro et al., 2013), and we clarified in the text that root-to-shoot translocation is driven by evapotranspiration and root pressure (line 377-378).

23. Line 459 „are key transporters“: Replace by „contribute to“. The phenotypes are not severe. It is quite possible, especially given the limitations of this study, that other transporters contribute to this, a conclusion that would also align with 10.1111/tpj.17251 (see also 10. above).

Modifications: **Modifications**: We have made the corresponding changes in the revised manuscript (line 526-527).

24. Lines 531-532: Include full description or a reference to a publication where this is fully described.

Response: Since the vectors are only partially published and lack a proper description, we now provide a detailed description in the Methods section. Additionally, we include a supplemental figure showing the maps of the two modified plasmids.

Modifications: We added the plasmid maps as Figure S11 and described them in the Methods section (line 627 and line 636).

Figure S11

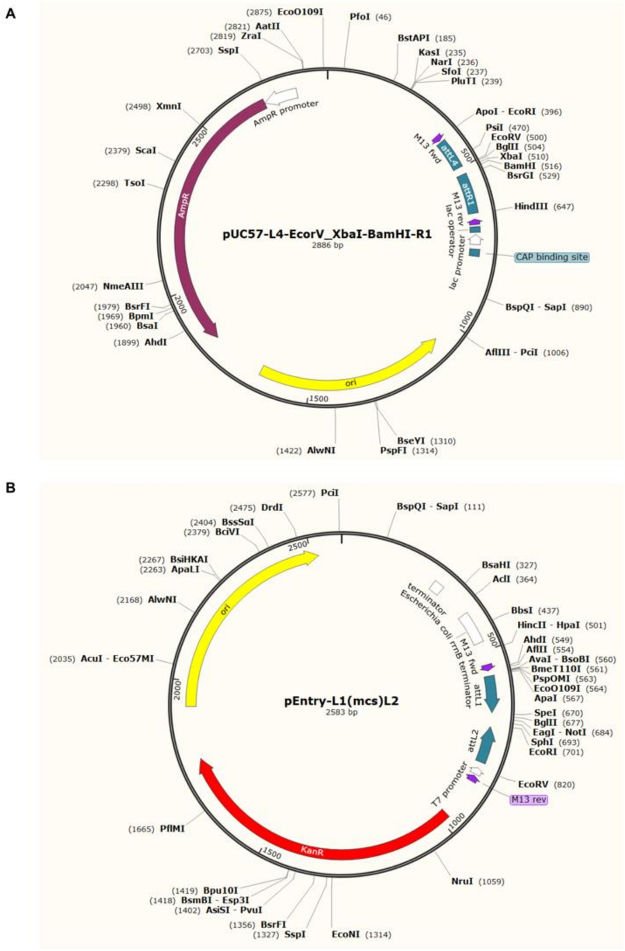

25. Lines 538-539: Please modify Fig. S2 to clarify the position of mCitrine, label TMH by numbers and clarify cytosolic side.

> **<u>Modifications</u>**: We revised the figure accordingly (now Figure S3) in the revised manuscript). Transmembrane helices (TMHs) are now indicated for the five presented ZIP proteins, along with annotations for the apoplastic and cytoplasmic sides, as well as the position of the mCitrine tag.

Figure S3

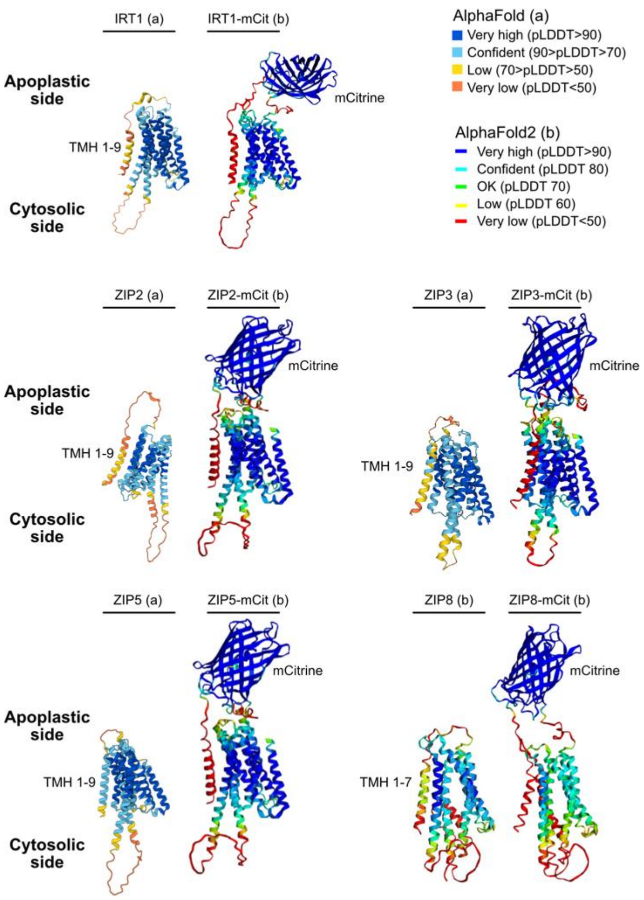

26. Lines 578: For all experiments on plates, add the number of seedlings per plate.

> **Modifications**: We have now included this information in the Materials and Methods section (line 675-677 of the revised manuscript).

27. Describe in methods the harvesting of materials from soil-grown plants, especially the harvesting and cleaning of roots for multi-element analysis (e.g. Fig. S7E).

> **Response:** We apologize for the omission. In the first version of the manuscript, we forgot to include a description of how soil-grown plants were harvested and washed. We have now updated the Materials and Methods section to provide this information.

> **Modifications:** A short section describing the harvesting and cleaning of roots for multi-element analysis has been added to the Materials and Methods (line 744-748 of the revised manuscript).

28. Line 580/81: Please clarify how these plants were grown, including number of seedlings per plate.

Modifications: We have now included this information in the Materials and Methods section (line 678-680 of the revised manuscript).

29. qRT-PCR lines 617-626. Please clarify the number of seedlings per plate and growth conditions. Please provide data on Clathrin control gene expression under the conditions employed here. Please provide data for primer pair amplification efficiencies.

Response: We verified primer efficiency, which ranged between 105% and 148% depending on the primer. As several primers showed efficiencies above 115%, we carefully checked the melting curves to confirm that only a single PCR product was amplified. These products were then sequenced, and for all primers used, sequencing confirmed that the amplified product corresponded to the intended target gene.

Modifications: We now provide detailed information on primer efficiencies in Table S4, and the Ct values obtained for Clathrin across different conditions and genotypes are listed in Table S5.

30. Please define what is meant by "biological replicates" in Line 626 and wherever else this term is mentioned in the manuscript – grown alongside on the same plate, on a different plate, or grown at a separate time (independent experiment?),...

Response: We used the term biological replicates to refer to independently collected pools of plants (ranging from a minimum of 3–4 plants to up to 40 seedlings, depending on the experiment, as specified in the figure legends). These pools were harvested from the same experiment, in which plants were grown and samples analyzed in parallel.

Modifications: We clarified this definition in the Materials and Methods section (line 805-809 of the revised manuscript).

31. Line 630: Please add the pH of the EDTA solution.

Response: The pH of the EDTA solution is adjusted to 5.7 using KOH.

Modifications: We clarified this information in the Materials and Methods section (line 743 of the revised manuscript).

32. ICP-OES: Please add instrument name/manufacturer, wavelengths, information on calibration (frequency) and QC.

Response: We contacted the platform where the ICP-OES analyses were performed to retrieve the requested information. We have now included the instrument name and manufacturer in the manuscript. However, despite several attempts, we were unable to obtain details regarding wavelength calibration frequency and quality control procedures

due to the unavailability of the responsible personnel in recent weeks. We will ensure that this information is included in the final version of the manuscript prior to publication.

Modifications: We added the instrument and manufacturer details in the Materials and Methods section (line 754).

33. Fig. 1D: Reviewer cannot find dashed line in the orange violin shape. Please check that all others are complete.

Response: We apologize for this oversight. The dashed lines in Figure 1 (now Figure S1) were indeed present but appeared in the same orange color as the background, making them impossible to see.

Modifications: We corrected the visibility of the dashed lines (in Figure S1B, S1C, and S1D in the revised manuscript). Additionally, we reviewed all figures in the manuscript to ensure that lines in violin plots are clearly visible throughout.

Figure S1

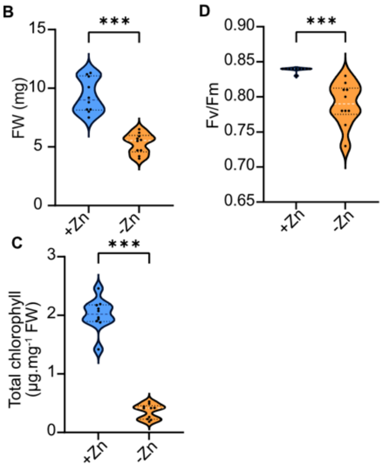

34. Fig. 4E: Change the use of axis breaks so that also Fe concentrations can be deciphered.

Response: Thank you for pointing this out. We agree that the axis break improves the readability of Fe concentration data. Additionally, in response to Reviewer 1's comment (see response to minor comment 1), we replaced the original Figure 4E with a revised figure (now Figure 3E), which presents data from two independent zip2 mutant lines.

Modifications: We now include both shoot and root Zn content for WT, *zip2-1* (T-DNA insertion line), and *zip2-2cr* (CRISPR mutant) in the same figure (now Figures 3E and 3F), and adjusted the axis break to enhance clarity.

Figure 3

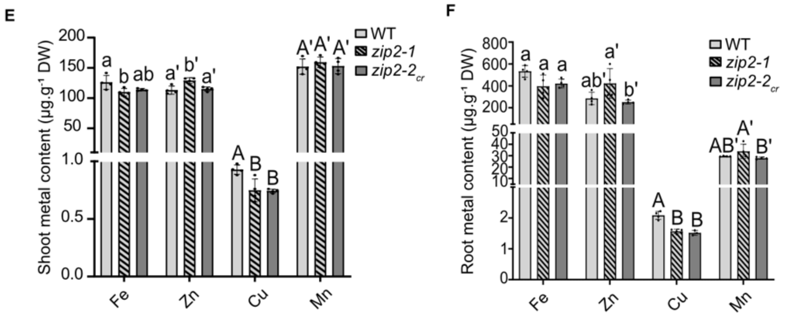

35. Fig. 5E: Please modify legend to explain the two WT in this figure.

Response: We included two WT samples because they originated from independent experiments; however, we agree that this may have been misleading for readers. This issue was also noted by Reviewer 1 (minor comment 2). The data presented in Figure 5E (now Figure 4F in the revised manuscript) were derived from two independent experiments. We recognize that the original figure layout may not have clearly conveyed this.

Modifications: We revised Figure 4F (formerly Figure 5F) to more clearly distinguish WT, *zip8-cr1*, and *zip8-cr2*, by increasing spacing and improving labeling. We also clarified this point in the figure legend to ensure better understanding of the experimental design.

Figure 4

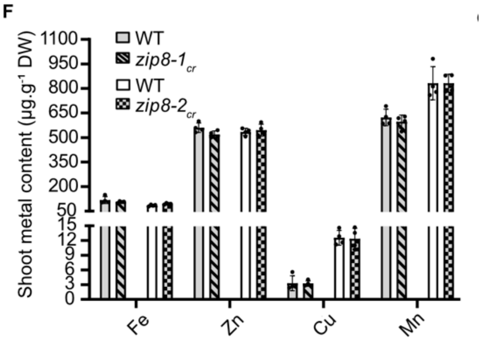

36. All vertical axis titles for root length: Please correct typo.

Modifications: All axis titles have been revised accordingly.

37. Why are Zn and Mn levels in Fig. S4I lower than elsewhere? Why are they lower in Table

S1? What was different from the other experiments? Please specify in methods and in the caption texts.

Response; We agree that Zn and Mn levels appear lower in Figure S4I (now Figure 3E) compared to other figures. We do not have a specific explanation for this, but we often observe variation in the basal levels of mineral accumulation between independent experiments, whether using ICP-OES or, in our previous studies, ICP-MS. Importantly, such variations do not affect the reproducibility of biological differences between genotypes or treatments.

We would also like to point out that in the same panel (Figure S4I), we provide ICP data for both WT and the *zip2-1* mutant, which show Zn and Mn levels comparable to other figures, and still demonstrate a reduction in Cu content in the mutant.

As for Table S1, the data correspond to plants grown on horizontal plates (as shown in the upper panel of Figure S1A). We now clarified this in the legend. We believe that this difference in growth orientation may explain variations in mineral levels compared to vertically grown plants, whose roots develop more extensively.

Modifications: We have clarified in the legend of Figure S1 how plants analyzed for ICP were grown.

38. It would be nice to see root data for Fig. S6 or S7 or another experiment in which Zn supply was not as excessive as on the agar-based media, also for zip3 (not only for zip5) and the zip3 zip5 double mutant.

Response; We agree that additional root data would be valuable for readers and would help strengthen our manuscript. In the revised version, we now provide ICP data for soil-grown *zip3*, *zip5*, and *zip3zip5* mutants, grown under conditions with lower Zn availability than our standard MS-based plates.

Additionally, we included visual phenotypes and root length measurements for the *ZIP2/3/5::IRT1* lines, all grown under both Zn-sufficient and Zn-deficient conditions.

Modifications: We added the ICP data as Figure 6E and the root data for the *ZIP::IRT1* lines as Figures S9A and S9B. The results section was adjusted accordingly in the revised manuscript (line 429-432).

Figure S9

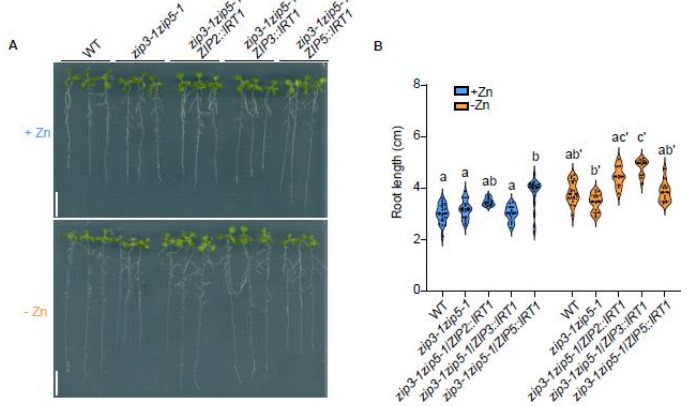

39. All experiments: The authors should provide ZT of harvest for all experiments involving plant growth in light-dark cycles.

Response: We agree with the reviewer that the Zeitgeber Time (ZT) of harvest is an important parameter, especially for experiments involving light/dark cycles.

Modifications: We have now clarified the ZT of harvest in the Materials and Methods section for all relevant experiments involving plants grown under light/dark conditions (line 677-678).

Reviewer #3:

Dear authors

The paper presents new data regarding as least two standing questions in the field: whether ZIP2 indeed transports copper, and which ZIPs are the primary zinc transporters for zinc uptake in arabidopsis. The data is clear and of high quality. My only major concern is with text organization: authors need to carefully separate sections in paragraphs, since many times the text is continuous even when topics change, including the introduction and results sections. Moreover, I suggest authors being more upfront about the recently published paper by Ochoa Tufiño in Plant Journal, which also demonstrates that ZIP3 and ZIP5 are zinc uptake transporters. Authors could compare and let readers know what is consistent, making a synthesis of both datasets. I believe this would not hurt the "novelty factor" of this work, but rather would make it even more valuable.

We thank Reviewer 3 for his very positive and encouraging assessment of our manuscript, as well as for the thoughtful and constructive comments. These suggestions have been extremely helpful in improving the clarity and quality of the manuscript. We address each of the points raised below.

Here are my other minor comments:

• Line 31: I think you can cite only as Palmer and Guerinot 2009?

Response: We agree that citing a single reference could have sufficed. However, in response to Reviewer 2’s suggestion, we added the citation of Zaal et al. (1999), and we chose to retain additional references as they provide a broader overview of the current knowledge on metal transporters.

Modifications: We did not implement the requested change.

• Line 43-44: “Cation Diffusion Facilitator (CDF) family, and the Metal Tolerance Proteins (MTP) family”. I think authors can mention only one of these families, as the CDFs are named MTPs in plants (i.e., they are not two distinct gene families as implied by the phrase).

Response: We agree with the reviewer's suggestion and have modified the manuscript accordingly.

Modifications: The text was revised as suggested (line 42).

• Line 53: please correct to "Oryza sativa" (sativa should be minor key, nor Sativa).

Modifications: The text was revised as suggested (line 70).

• Line 66-67: "Although AtIRT3 overexpression results in Zn and Fe accumulation" – please provide the reference for this.

Modifications: We added the reference Lin et al., 2009 (line 86).

• The paragraph from line 41 to 86 is very long and jumps from one topic to another. It needs careful reorganization. I suggest to make two paragraphs out of it; one more general about the ZIPs, an another about ZIP and Zn transports, mentioning the recently discovered function of OsZIP5/OsZIP9.

Response: We agree with the reviewer's suggestion.

Modifications: The text was revised as suggested.

• Line 108: I suggest changing "the plants stunted growth and chlorosis" to "the plants showed stunted growth and chlorosis" or similar.

Response: We agree with the reviewer's suggestion.

Modifications: The text was revised as suggested (line 123-124)

• Line 138: I am aware that the authors briefly mention the recently published paper bu Ochoa Tufino in Plant Journal in their discussion. I also understand that this paper came out before and authors only became aware of it after finishing their own work. However, I think the community would benefit from a comparison of gene expression results of both papers. Authors should consider performing that, so readers can see what is consistent.

Response: We agree with the reviewer. The study by Ochoa Tufiño et al. (2025) was published after our manuscript was deposited on bioRxiv. While we had cited it in our initial submission, we did not include a detailed comparison of our data with theirs. As suggested, we have now integrated such a comparison in the revised manuscript, which we believe strengthens our results and provides valuable context. In particular, we highlight that our data support partial rather than complete redundancy between ZIP3 and ZIP5, in contrast to the conclusions drawn by Ochoa Tufiño et al. (2025), and we discuss possible explanations for these differences.

Regarding gene expression, we chose not to include *ZIP* expression comparisons in WT plants under +Zn and -Zn conditions (Figure 1B), as these have been previously reported in several studies. However, we now briefly comment in the discussion on the similarities in gene expression observed in the double *zip3zip5* mutant (Figure S8).Also, as reviewer 2 noticed, an error in ZIP4 and ZIP6 expression was present. We now fully corrected this Figure 1B and Figure S8B.

Modifications: We added several comparisons and interpretations related to the work by Ochoa Tufiño et al. (2025) in the discussion (lines 523–548, 553–556, and 567–570).

Figure 1

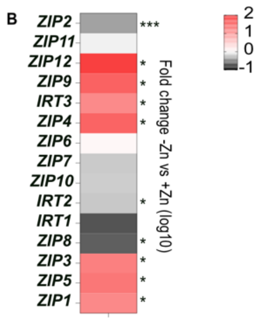

Figure S8

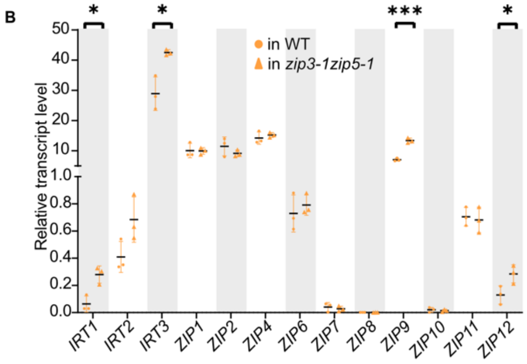

• Line 149: this could the start of a new paragraph, to improve the flow of the text: "Anticipating that transporters…"

Response: We agree with the reviewer's suggestion.

Modifications: We modified the text accordingly (line 164)

• Line 169-170: I understand that this is possible, but it's highly speculative based solely on this data. I suggest suppressing it here and moving to the discussion sections, making sure to highlight that it's only a speculation.

Response: We fully agree with the reviewer's comment — this part is indeed speculative and not supported by data presented in the manuscript, and therefore does not belong in the Results section.

Modifications: We removed this statement from the revised text.

• Line 170: "Based on our expression analysis" could be the start of a new paragraph. Also, I think it's "analyses", since you performed several, not only one.

Response: We agree with the reviewer's suggestion and have implemented the proposed change in the revised manuscript.

Modifications: We modified the text accordingly (line 195).

• General comment: the text needs structural revisions, since often there is not paragraph separation. Revise throughout.

Response: We agree with the reviewer's comment. We revised the text to improve clarity by restructuring the section, dividing it into more paragraphs, and reorganizing the figures to better align with the narrative.

Modifications: We modified the text accordingly, improved the structure by separating it into clearer paragraphs, and reorganized the corresponding figures.

• Line 289: "both zip8 alleles" sounds like colloquial lab language. We use it, but in the paper I suggest using "both zip8 mutant lines".

Response: We agree with the reviewer's comment and appreciate the suggestion.

Modifications: We have revised the manuscript accordingly (line 317-318).

• Line 309: I think the claim "…does not fully overlap with IRT1's function" is not supported by the evidence. Although compelling to claim that ZIP8 is indeed involved somehow in Fe acquisition, whether that overlaps with IRT1 would need double mutants and gene swap experiments, for example. I think authors should suppress it, or move it to the discussion section.

Response: We agree with the reviewer's comment. Our data do not support the claim that ZIP8 function does not fully overlap with that of IRT1, and we acknowledge that this conclusion was not sufficiently substantiated.

Modifications: We have removed this statement from the manuscript.

• Figure 6 and data therein: could authors provide a clearer/closer image of plant shoots under +Zn and -Zn. Despite the chlorophyll measurement, it would be key to see what is the visual phenotype I shoots of the double mutant zip3zip5 under -Zn. Since this is one of the key finding of the paper, I strongly suggest authors to provide that.

Response: We agree with the reviewer's comment and now provide zoomed images of WT and *zip3zip5* mutant shoots grown under both +Zn and –Zn conditions to better illustrate their phenotypes. In addition, we included new data on *zip3zip5* at 20 days after

germination on Zn-sufficient and Zn-deficient media. We also measured shoot fresh weight and chlorophyll content at this later stage to further support our observations.

Modifications: We added zoomed shoot images of WT and *zip3-1zip5-1* plants as Figure 5B, and we now provide phenotypic, chlorophyll, and fresh weight data for 20-day-old WT, *zip3-1*, *zip5*, and *zip3-1zip5-1* plants as Figures S7F–H). The results text has been revised accordingly (line 360-366).

Figure 5

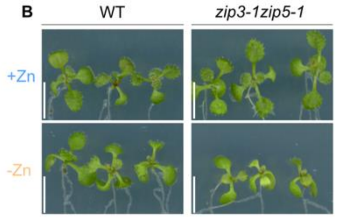

Figure S7

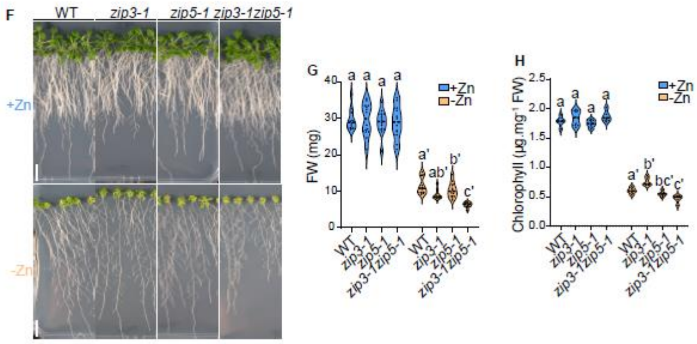

• I suggest inverting figure 6G to match the common orientation used in the paper.

Response: We appreciate the reviewer's suggestion. For space optimization, we have opted to retain its current orientation, which we believe allows for better integration with the surrounding panels and overall figure design.

• Line 333-335: why there is not ICP data for the single mutants?

Response: The ICP data for the single mutants were originally provided in Figure S7 (first version of the manuscript). We apologize if they were not easily located. To improve clarity and accessibility of the data, especially regarding ICP, we have made substantial changes to the figure layout, as detailed below.

Modifications: To clarify the presentation and interpretation of the data, we split the original Figure 6 into three main figures (now Figures 5, 6, and 7).

- The new Figure 5 focuses on the phenotypes of WT, the single mutants *zip3-1* and *zip5-1*, and the double mutant *zip3-1 zip5-1*. Additional phenotypic data for these and other alleles are provided in Figure S7.

Figure 5

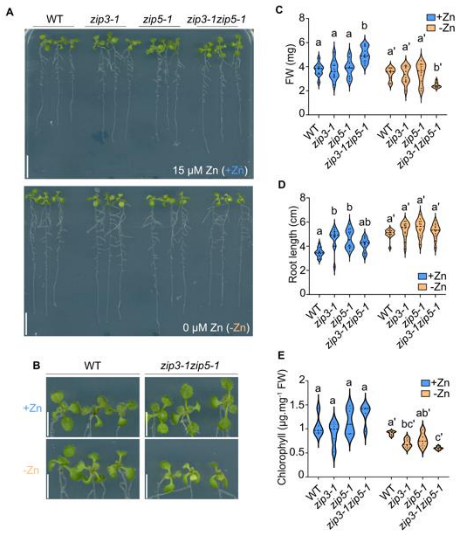

- The central ionomic data, including ICP measurements for single and double mutants, are now consolidated in Figure 6. This includes data for a second *zip3 zip5* allele as well as data from soil-grown plants, where Zn levels are considerably lower than on agar plates. Figure S8 complements Figure 6 by providing additional ICP data for the single mutants.

Figure 6

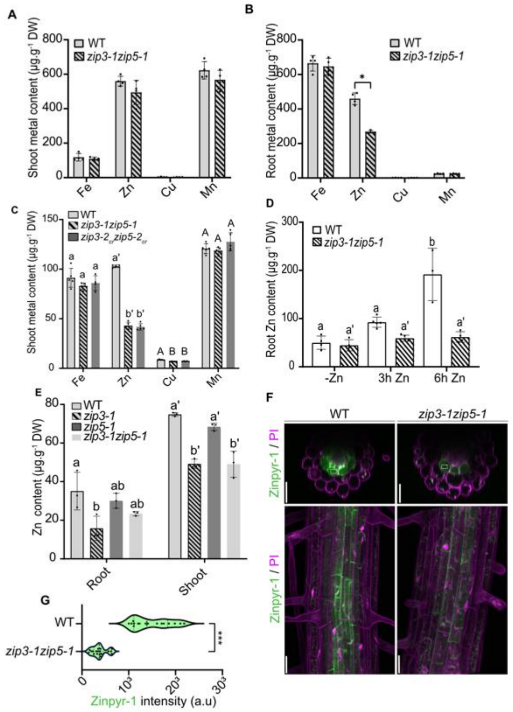

- The new Figure 7 presents data on overexpression lines and the role of IRT1 in Zn transport (previously in Figure S7G-S7H). A new supplementary figure (Figure S9) linked to Figure 8 shows root phenotypes of the *ZIP::IRT1* lines grown under both Zn-sufficient and Zn-deficient conditions.

Figure 7

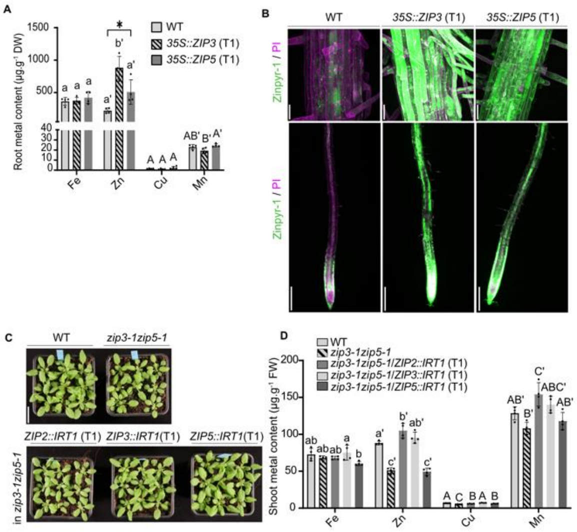

- The corresponding text in the Results section has been revised accordingly.

- In this section of the paper the lack of proper organization in paragraphs is very clear. Authors need to carefully revise that. It does not affect the content quality of the manuscript, but should be reorganized.

Response: We agree with the reviewer that this section of the manuscript would benefit from better organization and clearer structure. A clearer separation between paragraphs improves readability and helps convey the key points more effectively.

Modifications: We have reorganized this section by dividing the text into several shorter paragraphs to improve clarity and facilitate comprehension.

• Line 345: “shoot analysis revealed that ZIP3 is primary responsible”. This is based on the observation that zip3 mutants have lower Zn concentration? I think authors could be a bit less strong about it, since this suggest ZIP3 has a primary role over ZIP5.

Response: We speculated that ZIP3 plays a predominant role over ZIP5 because the zip5 single mutant does not exhibit a clear Zn accumulation defect. However, we agree with the reviewer that we could have been more explicit in our conclusion. We have now clarified in the revised text that the lack of phenotype in zip5, combined with the detectable Zn phenotype in zip3, supports the idea that ZIP3 and ZIP5 function only partially redundantly, with ZIP3 likely playing a more prominent role in Zn acquisition.

Modifications: We have revised the manuscript accordingly to reflect this interpretation (line 398-404).

• Why the ICP data on singles and doubles not presente together. I think it should be in the main figures.

Response: We agree with the reviewer's comment, and as mentioned in our response to the comment on lines 333–335 regarding the single mutants, ICP data for these lines were included in Figure S7 of the first version of the manuscript. We apologize if they were not easily accessible. To improve clarity and facilitate interpretation, we reorganized the figures and presentation of the data, as described above.

Modifications: As detailed above, we reorganized the data presentation and added ICP data for both single and double mutants into the main figures. These are now shown in Figure 6, which focuses on the phenotypic and ionomic characterization of *zip3*, *zip5*, and *zip3 zip5* mutants.

Figure 6

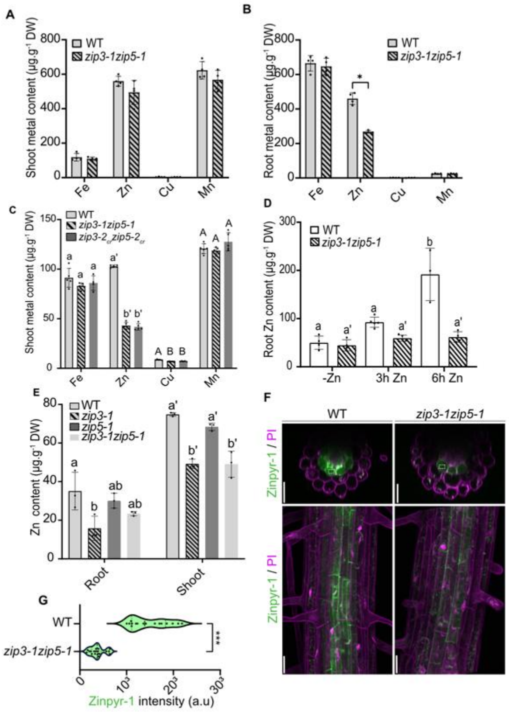

• Line 351: "To gain further insight..." is a clear new paragraph, as an example to help authors organize the text.

Modifications: We have made the corresponding changes in the revised manuscript (line 407).

• Line 463-464: please correct to "Zn uptake IN root".

Modifications: We have made the corresponding changes in the revised manuscript (line 533).
