# Supplementary material for: Four ZIPs contribute to Zn, Fe, Cu and Mn acquisition at the outer root domain
Source: PLoS Genet. 2025 Jul 15;21(7):e1011796. doi: 10.1371/journal.pgen.1011796 (PMC12279101; doi:10.1371/journal.pgen.1011796)
Supplement: Attachment — Submitted filename: Response to reviewer.docx [file pgen.1011796.s019.docx]

Dear Editors and Reviewers,

We sincerely thank you and the reviewers for your positive evaluation of our revised manuscript entitled *"Four ZIPs contribute to Zn, Fe, Cu and Mn acquisition at the outer root domain"*. We are pleased to hear that the manuscript is provisionally accepted for publication in *PLOS Genetics*.

We have carefully considered the remaining minor comments from Reviewer 2 and have made the necessary changes to the manuscript, as detailed in the point-by-point response below. All modifications are clearly marked in the revised version of the manuscript.

Thank you again for your consideration

Below, you will find our detailed point-by-point responses to the reviewer’s comments.

Our responses are marked in green, and modifications to the manuscript are indicated in blue.

With our best regards.

Marie Barberon and Kevin Robe

**Reviewer #1:** The revised version of the manuscript shows significant improvement compared to the previous submission. The authors have carefully addressed all the major concerns I raised in my initial review. The clarification and additional data provided have strengthened the overall quality and clarify the manuscript. I have no additional comments at this time.

We thank the reviewer for the positive feedback and thoughtful evaluation of our revised manuscript. We are pleased to hear that the revisions and additional data have clarified the points previously raised and improved the overall quality of the work. We greatly appreciate the reviewer’s time and support throughout the review process

**Reviewer #2:** In their first revision, the authors have adequately addressed most of the issues I had raised. Only a few minor aspects are remaining and need some additional attention or modifications (see below).

We thank the reviewer for their constructive feedback and for acknowledging that most of the issues raised have been adequately addressed in our previous revision. We appreciate the careful reading of our manuscript and the helpful suggestions. We have now carefully considered the remaining minor points and have made the necessary modifications as detailed below. We also added information on the ICP measurement as asked in the previous review.

1. Fig. 1B labeling Log10(fold change -Zn vs. + Zn), and this text would be better placed next to the legend relating numbers to colors (or explain in caption text). After describing the results shown in this panel in the results text, the authors should cite the publications in which the upregulation of transcript levels of each of the respective ZIPs under Zn deficiency was first reported in roots of Arabidopsis thaliana (e.g. partly Talke et al., 2006; Assuncao et al., 2010; Sinclair et al., 2018, ..., for example).

**Modifications**: we modified accordingly Figure 1B and added references in the text. (line 160)

2. Caption text Fig. 1C: “a single focal plane”; please add here that seedlings were grown under control conditions (age would be useful, too).

**Modifications**: we added the age of the seedlings in the legend, and the control growth conditions in the legend of Figure 1. (line 1099)

3. Fig. 3A: The left panel says –Cu, but most likely the agar contained some Cu so that the total Cu level will not be zero. The authors should make this more explicit somewhere (methods).

**Modifications**: we mentioned in the material and methods section that trace amounts of metals are likely still present even in -metal condition. (Line 678-679)

4. Fig. 4, caption text, final line “obtained”.

**Modifications**: we modified the text legend accordingly (line 1163)

5. L. 68: “vacuolar Zn storage”

**Modifications**: we modified accordingly (line 64)

6. L. 300: suggest to replace “critical role for ZIP2” by “contribution of ZP to”, given the extremely low Cu levels even in wild-type in plants under the –Cu conditions used here and the quantitatively only small relate difference between zip2 mutants and the wild type.

**Modifications**: we modified accordingly (line 282)

7. Line 309: Similarly (see 9.), suggest to delete “major” in line 309.

**Modifications**: we modified accordingly (line 291)

8. Line 317: Suggest to replace “in the root” by “into the root”

**Modifications**: we modified accordingly (line 296)

9. Line 335: “ a truncated peptide of 2 aa in length”; add length of predicted produced peptide also for zip8-2.

**Modifications**: we added this information (line 314)

10. Line 351 “of both zip8 mutant lines”

**Modifications**: we modified accordingly (line 319)

11. Line 406: On agar plates, rosette tissues may absorb or take up metals from the medium. This may explain the comparably high metal levels in above-ground tissues, and it is not possible in soil-grown plants. This could also be added when discussing these observations.

**Response**: We thank the reviewer for this valuable suggestion. While we acknowledge that rosette tissues grown on agar plates may absorb metals directly from the medium, potentially contributing to elevated metal levels in above-ground tissues, we chose not to include this hypothesis in the discussion to avoid overcomplicating the interpretation of our results. Not all mutants analyzed in this study exhibited reduced metal accumulation in leaves when grown on agar. For instance, *zip2* mutant leaves showed reduced copper accumulation even on plates, whereas *zip8* and *zip3zip5* mutants did not display significantly altered metal content. Including this alternative explanation could therefore create confusion, as it may not uniformly apply across all genotypes studied.

12. Lines 413-417: Given that root-to-shoot Zn translocation ratios are similar in zip3 zip5 and WT (Fig. S8A), the reviewer is not sure about the role proposed here of other ZIPs in root-to-shoot Zn translocation in the zip3 zip5 double mutant. The authors explain their thoughts a little better in the discussion lines 608-611, and maybe this text should be moved there from the results.

what the authors mean here – it would be better to rephrase this sentence.

**Response**: We would like to clarify our idea here. Based on ZIP5 and ZIP3 localization in the stele, we hypothesized that these two ZIP are involved somehow in Zn partitioning between root and shoot. Yet, since we could not observed Zn translocation defect in the double mutant, we wondered if other ZIP might take over this function in the stele. The upregulation of some ZIPs in the *zip3zip5* double mutant could reflect this role. Yet, it remains speculative at this stage and is not supported by functional data.

**Modifications**: we clarified our hypothesis on the proposed role of other ZIPs in root to shoot Zn translocation in the *zip3zip5* double mutant. (line 385-388)

13. Line 423: “reduction” reads like efflux of Zn from the roots of the double mutant, which is not what the authors mean here – it would be better to rephrase this sentence.

Response: we agree with reviewer comment.

**Modifications**: we slightly modified the text to clarify it. (line 394-395)

14. Line 569: The authors should specify what they mean by “this function” here.

**Modifications**: we now specified in the text that we are referring to its function of Fe acquisition from the soil. (line 524)

15. Line 575: “are closer to”. The authors should write more precisely what they mean by this.

**Modifications**: we modified the text to mention that ZIP1 and ZIP12 “show higher sequence similarity” instead of “are closer”, since the phylogenetic tree in Zheng et al., 2018 is based on sequence. (line 530-531)

16. Line 594: “in this point of view” – the authors should state more precisely what they mean by this (e.g. which point of view).

**Response**: we apologize for this “frenglish”.

**Modifications**: We modified to be clearer. (line 547)

17. There are lacking spaces in some positions in the manuscript text, and also some grammatical errors, typos, formatting issues (references) and stylistic issues (e.g. first words identical in lines 612 and 620), especially in the methods.

**Modifications**: We modified line 567 for stylistic issues and checked all references. We removed a duplicate in the bibliography (Milner et al., 2014) and some additional typos we identified.

18. Line 634: The reviewer feels that this text needs a few additions. The authors should note that MTP2 transcript is only present in highly Zn-deficient plants, and a symplastic transport of Zn2+ from cell to cell via plasmodesmata is not only possible via the ER but also within the cytosolic regions surrounding the desmotubule. The authors should also consider that Zn transport could be entirely symplastic after uptake in the outer cell layers, and additional Zn uptake could occur into endodermal cells without any Zn efflux on the inside of cortex cells (the presence of an uptake system on the endodermis cannot provide evidence for efflux from outer cell layers of the root).

**Response**: We agree with the reviewer’s comment that symplastic Zn transport do not necessarily rely on MTP2, especially considering that MTP2 is only expressed in Zn deficiency. Yet, we propose different and not mutually exclusive scenario for Zn radial transport in the discussion section, highlighting the putative involvement of different ZIP for endodermal Zn uptake or symplastic transport.

**Modifications**: We modified the text to propose that Zn can be transported through cytosolic sleeves of plasmodesmata and possibly via the ER localized MTP2 in some conditions. (line 578-582)

19. Methods: Precise PCR conditions and other methodology for obtaining DNA fragments, cloning procedures with appropriate literature references (including also the origin of the mCitrine) should be specified in detail.

**Modifications**: we added some details on PCR conditions in the material and methods section. (Line 627-629) and a reference for the mCitrine (Dubeaux et al., 2018, line 644).

20. Methods: The authors should specify the type (shape, dimensions) of agar plates used for plant cultivation and the volume of medium per plate.

**Response**: We agree that this information is important.

**Modifications:** We clarified line x the density of sowing, the size of plate and the volume of media used. (line 672-674)

21. Methods: It appears that many experiments were done in T1. The authors should specify how and when they selected transgenics in the experiments described in lines 725-752.

**Modifications:** We clarified how T1 seeds were selected from the experiments line (line 730-731)

22. Lines 792-3: Sentence needs fixing (redundancies).

**Modifications:** We modified the text. (line 738-740)

23. Statistics: It would be good to know how many independent replicate agar plates were reflected (approximately) among all biological replicates.

**Modifications:** We clarified line 672-674 the density of sowing for ICP and expression analysis. Number of biological samples per replicate for each experiment is indicated in the legend of each figure.

24. Authors should indicate at which stage plasmids, constructs or inserts were sequenced to verify, the extent, and the technique used.

**Modifications:** we added these informations. (line 661-662)

**Reviewer #3**: The authors have adequately improved the manuscript.

We thank the reviewer for the positive feedback and thoughtful evaluation of our revised manuscript. We are pleased to hear that the revisions and additional data have clarified the points previously raised and improved the overall quality of the work. We greatly appreciate the reviewer’s time and support throughout the review process
